# Senolytic effect of triterpenoid complex from *Ganoderma lucidum* on adriamycin-induced senescent human hepatocellular carcinoma cells model *in vitro* and *in vivo*

**DOI:** 10.3389/fphar.2024.1422363

**Published:** 2024-09-19

**Authors:** Ahmed Attia Ahmed Abdelmoaty, Jing Chen, Kun Zhang, Changhui Wu, Ye Li, Peng Li, Jianhua Xu

**Affiliations:** ^1^ Fujian Provincial Key Laboratory of Pharmacology of Natural Medicine, School of Pharmacy, Fujian Medical University, Fuzhou, China; ^2^ Fujian Xianzhilou Biological Science and Technology Co., Ltd., Fuzhou, China

**Keywords:** hepatocellular carcinoma, triterpenoid complex, *Ganoderma lucidum*, senescence, senolytic agent

## Abstract

**Background:**

*Ganoderma lucidum* (*G. lucidum*) is a famous medicinal mushroom that has been reported to prevent and treat a variety of diseases. Different extractions from *G. lucidum* have been used to manage age-related diseases, including cancer. Nevertheless, the senolytic activity of *G. lucidum* against senescent cancer cells has not been investigated. Although cellular senescence causes tumor growth inhibition, senescent cells promote the growth of the neighboring tumor cells through paracrine effects. Therefore, the elimination of senescent cells is a new strategy for cancer treatment.

**Methods:**

In this study, senescence was triggered in HCC cells by the chemotherapeutic agent Adriamycin (ADR), and subsequently, cells were treated with TC to assess its senolytic activity.

**Results:**

We found for the first time that the triterpenoid complex (TC) from *G. lucidum* had senolytic effect, which could selectively eliminate adriamycin (ADR)-induced senescent cells (SCs) of hepatocellular carcinoma (HCC) cells via caspase-dependent and mitochondrial pathways-mediated apoptosis and reduce the levels of senescence markers, thereby inhibiting the progression of cancers caused by SCs. TC could block autophagy at the late stage in SCs, resulting in a significant activation of TC-induced apoptosis. Furthermore, TC inhibited the senescence-associated secretory phenotype (SASP) in SCs through the inhibition of NF-κB, TFEB, P38, ERK, and mTOR signaling pathways and reducing the number of SCs. Sequential administration of ADR and TC *in vivo* significantly reduced tumor growth and reversed the toxicity of ADR.

**Conclusion:**

A triterpenoid complex isolated from *G. lucidum* may serve as a novel senolytic agent against SCs, and its combination with chemotherapeutic agents may enhance their antitumor efficacy.

## 1 Introduction

Hepatocellular carcinoma (HCC) is one of the most prevalent malignant tumors in China and the third leading cause of cancer mortality in the world (Tang, 2001; Parkin et al., 2005). Due to the limitations of the treatment strategies available for HCC (Bartlett and Heaton, 2008; Xu et al., 2014), novel therapeutic options are required to reduce the incidence and mortality associated with HCC.

Cellular senescence is a biological process that shares some common features, including irreversible cell cycle arrest, increasing the activity of senescence-associated-β-galactosidase (SA-β-gal), relative resistance to apoptosis, persistent DNA damage signaling accompanied by γ-H2AX foci formation, and upregulation of the cell cycle inhibitors p21^Cip1/Waf1^, p16^Ink4a^ and p53 (Braig et al., 2005; Collado et al., 2005). Although cellular senescence is considered as a tumor suppression mechanism, senescent cells secrete many factors, including cytokines, proteases, growth factors, and chemokines termed as SASP, which promotes the malignant phenotype and resistance to treatment in residual tumor cells through paracrine effects, thereby stimulating tumor recurrence and metastasis (Zhang et al., 2005; Childs et al., 2015). Since senescent cells set up a microenvironment conducive to tumor development and treatment resistance, they become a new hallmark of cancer (Hanahan, 2022). Senescence happens after treatment with radiation and/or specific chemotherapies, known as therapy-induced senescence (TIS) (Ewald et al., 2010). Some cancer cells reprogrammed by TIS to gain a change in stemness can develop into self-renewing tumor-initiating cells that induce aggressive growth and tumor recurrence (Cahu et al., 2012). Therefore, senotherapeutics targeting senescent cells have become a new strategy for cancer treatment. They include senomorphics (small molecules that partially inhibit senescence phenotypes, such as the SASP, without killing senescent cells) and senolytics (small molecules that cause the death of senescent cells) (Qu et al., 2013; Lu et al., 2014).

Adriamycin is the first-line chemotherapy agent for transarterial chemoembolization and is widely used for advanced-stage HCC (Wang et al., 2024). However, adriamycin can induce senescence in both normal and tumor cells, which may contribute to therapeutic resistance, tumor recurrence, and adverse reactions, including severe cardio, hematological, renal, and hepatic toxicities (Lerida-Viso et al., 2022; Kciuk et al., 2023). As with cancer cells, senescent cells have the ability to upregulate various pro-survival and anti-apoptotic signaling pathways, such as PI3K/Akt, Bcl-2/Bcl-xl, and initiate autophagy (Goehe et al., 2012; Liu et al., 2020). The cocktail of the drugs dasatinib and quercetin (D + Q) can potentially target a number of these pro-survival pathways and modulate senescence in liver cancer (Li et al., 2020). However, D + Q combination was ineffective against adriamycin-induced senescent HCC cells because the upregulation of anti-apoptotic/pro-survival pathways in senescent cells varies depending on cellular senescence induction methods (Hernandez-Segura et al., 2017; Kovacovicova et al., 2018). Therefore, it is critical to find molecules that selectively eliminate therapy-induced senescent HCC cells as a potential treatment for liver cancer.

*G. lucidum* is a popular traditional Chinese medicine known as “Ling Zhi” in China (Wu et al., 2017). It has long been utilized as a traditional medicine for improving health (de Mattos-Shipley et al., 2016). *G. lucidum* contains different bioactive compounds, including polysaccharides, triterpenes, amino acids, proteins, enzymes, vitamins, flavonoids, alkaloids, minerals, and steroids. Among these components, polysaccharides and triterpenes are the major pharmacologically active compounds of *G. lucidum* fruiting body (Paterson, 2006). *G. lucidum* has been found to have various pharmacological actions, such as anti-cancer (Guo et al., 2018; Zhao et al., 2018), immunomodulator (Ishimoto et al., 2017), antioxidant (Kan et al., 2015; Bhardwaj et al., 2016), antimicrobial (Gaylan et al., 2018), anti-inflammatory (Cai et al., 2016), and hepatoprotective effects (Chatterjee and Acharya, 2016). To date, polysaccharides, triterpenes, and peptides of *G. lucidum* have been reported to have anti-aging properties (Wang et al., 2017). However, no studies have demonstrated the ability of *G. lucidum* to selectively eliminate senescent cancer cells.

This recent work is the first to reveal the senolytic effect of a specific active triterpene complex (TC) from the fruiting body of *G. lucidum* on ADR-induced senescent human hepatocellular carcinoma cells model *in vitro* and *in vivo*. Our findings indicate that TC selectively eliminates SCs by inducing apoptosis, which is associated with the downregulation of the anti-apoptotic Bcl-2 family members (Bcl-xl, Bcl-2, and Bcl-w), and the pro-survival PI3K/AKT pathway. Moreover, TC blocks autophagy at the late stage and inhibits the SASP in SCs. Therefore, TC emerges as a promising senolytic agent for targeting therapy-induced senescent HCC cells.

## 2 Materials and methods

### 2.1 Regents and antibodies

TC fraction was extracted in our laboratory, and a 50 mg/mL stock solution was prepared in dimethylsulfoxide (DMSO). Navitoclax (ABT-263) was purchased from MedChemExpress. Chloroquine, bafilomycin A1, and rapamycin were purchased from Aladdin (Shanghai, China). Antibodies against β-Actin, p21^Cip1/Waf1^, Bcl-xl, Bcl-2, Bcl-w, caspase-3, cleaved caspase-3, caspase-7, cleaved caspase-7, caspase-8, cleaved caspase-8, caspase-9, cleaved caspase-9, PARP, cleaved PARP, Akt, P-Akt, LC3 I/II, P62, NF-κB, P-NF-κB, TFEB, P38, P-p38, Erk, P-Erk, mTOR, and P-mTOR were purchased from Cell Signaling Technology (Danvers, MA, United States). Antibodies against p16^Ink4a^ and γ-H2AX were purchased from Abcam (Cambridge, UK). The rest of the chemical reagents were of analytical grade.

### 2.2 Sample preparation and analysis

The dry powder from the fruiting body of *G. lucidum* was extracted three times by reflux with 90% EtOH, each time for 3 h. The entire extraction solution was concentrated under reduced pressure and dried under a vacuum to obtain *G. lucidum* EtOH extract (GLE). The GLE was redissolved in 60% ethanol and then loaded onto the top of a glass column wet-packed with the AB-8 macroporous adsorption resin. The feed rate was 1 BV/h (BV standing for bed volume), and the eluting solvent flow rate was 2 BV/h. After loading, desorption was successively carried out with ethanol concentrations of 60% and 95%. The elution volume was kept at 6 BV for each concentration of ethanol. To obtain TC, the elution of 95% ethanol was concentrated under reduced pressure and dried under a vacuum. The total triterpenoid contents of TC and GLE were measured using Varian Cary 50 ultraviolet-visible spectrophotometry with oleanolic acid as a reference standard, and the total triterpenoid content was represented as an oleanolic acid equivalent through the calibration curve for oleanolic acid. The HPLC analysis of TC, GLE, and ganoderic acid A was determined according to our previous report (Zhu L. et al., 2020). The samples were dissolved in chromatographic grade acetonitrile solution and then filtered using 0.45 μm microporous membrane. 20 μL supernatant was injected into the HPLC system. Chromatographic conditions: LC-20AD liquid chromatograph (Shimadzu Corporation, Kyoto, Japan) with Kromasil C18 column (4.6 × 250 mm, 5 μm). Mobile phase: acetonitrile (B) - 2% acetic acid water (A), gradient mode: 0 min, A: 65%, B: 35%; 10 min, A: 63%, B: 37%; 30 min, A: 61%, B: 39%; 35 min, A: 55%, B: 45%; 50 min, A: 38%, B: 62%; 90 min, A: 0%, B: 100%. The flow rate was 1.0 mL/min. The column temperature was 30°C. The detection wavelength was 254 nm. After that, TC, ganodermanondiol, ganodermanontriol, ganoderiol A, ganoderiol B, and ganoderal A were analyzed via optimized HPLC chromatographic conditions: Mobile phase: acetonitrile (B) - water (A), gradient mode: 0 min, A: 50%, B: 50%; 40 min, A: 0%, B: 100%; 50 min, A: 0%, B: 100%.

### 2.3 Cell culture and treatment

Human HepG2 and Sk-Hep-1 cells were cultured in Dulbecco’s modified Eagle medium (DMEM) supplemented with 10% fetal bovine serum (FBS) and 1% penicillin-streptomycin in a humidified incubator with 5% CO_2_ at 37°C. For induction of senescence, 24 h after incubation, the cells were treated with 0.125 μg/mL ADR (Sigma-Aldrich) or DMSO as a vehicle control for 3 days before analyses. ADR treatment was followed by TC (100 and 150 μg/mL), 5 μM z-VAD-fmk, 15 μM navitoclax, 20 μM chloroquine, 25 nM bafilomycin A1, or 10 μM rapamycin.

### 2.4 Cell viability assays and senolytic index calculation

For HepG2 and Sk-Hep-1 cell lines, non-senescent cells (NCs) and SCs were cultured in 96-well plates in triplicate at a density of 4 × 10^3^ cells/well and incubated with vehicle (0.1% DMSO) or various concentrations of navitoclax and TC for 48 h. Cell viability was assessed by MTT assay (Xu et al., 2018). The half-maximal inhibitory concentration (IC50) was calculated to evaluate the cytotoxicity of navitoclax and TC on NCs and SCs. Senolytic indices were calculated by the IC50 of TC in NCs divided by the IC50 in SCs, which can be used as an indicator of senescence-selectivity.

### 2.5 SA-β-gal staining

To quantify senescence, treated cells were washed using 1x phosphate-buffered saline (PBS) and fixed with the fixative solution at room temperature for 15 min. The cells were then washed three times with 1× PBS and stained with 1× β-gal staining solution according to the manufacturer’s instructions. The cells were incubated for 24–48 h in a 37°C incubator without CO_2._ The cells were analyzed with a light microscope. For SA-β-Gal staining in tumor samples, tumors were fixed with 4% paraformaldehyde overnight at room temperature, washed three times with PBS, and incubated with 1x β-gal staining solution at 37°C for 6 h.

### 2.6 Quantitative real-time PCR

Total RNA was extracted from both control and treated cells with TRIzol reagent (Ambion, United States). The reverse transcription (RT) of RNA into cDNA was performed using HiScript III (+gDNA wiper) reverse transcription kit (Vazyme, China). Quantitative real-time reverse transcription polymerase chain reaction (qRT-PCR) was carried out using ChamQ SYBR qPCR Master Mix (Vazyme, China), according to the manufacturer’s protocol. For qRT-PCR analysis, the following primers were used: p21^Cip1/Waf1^ (forward 5′-TGT CCG TCA GAA CCC ATG CG-3′ and reverse 5′-AAA GTC GAA GTT CCA TCG CTC-3′), p16^Ink4a^ (forward 5′-ACT ATT CGG TGC GTT GGG CA-3′ and reverse 5′-AGC ATG GAG CCT TCG GCT GA-3′), IL-6 (forward 5′-ACT CAC CTC TTC AGA ACG AAT TG-3′ and reverse 5′-AGC CAT CTT TGG AAG GTT CAG-3′), IL-1β (forward 5′-GCA​CTA​CAG​GCT​CCG​AGA​TGA​A-3′ and reverse 5′-GTC​GTT​GCT​TGG​TTC​TCC​TTG​T-3′), IL-1α (forward 5′-GCA​TGC​CAT​CAC​ACC​TAG​TT-3′ and reverse 5′-TTA​CAT​ATG​AGC​CTT​CAA​TG-3′), Human NF-κB p65 (forward 5ʹ-AGG​CAA​GGA​ATA​ATG​CTG​TCC​TG-3ʹ and reverse 5ʹ-ATC​ATT​CTC​TAG​TGT​CTG​GTT​GG-3ʹ), Human TFEB (forward 5′-CCA​GAA​GCG​AGA​GCT​CAC​AGA​T-3′ and reverse 5ʹ-TGT​GAT​TGT​CTT​TCT​TCT​GCC​G-3ʹ) and GAPDH (forward 5′-AGA AGG CTG GGG CTC ATT TG-3′ and reverse 5′-AGG GGC CAT CCA CAG TCT TC-3′).

### 2.7 Apoptosis assay

Apoptosis was measured using Annexin V-APC Apoptosis Detection Kit (Elabscience Biotechnology, China), according to the manufacturer’s instructions. Control and treated cells were harvested by trypsinization, centrifuged, and washed once with PBS. Then, the cells were suspended in 100 μL of binding buffer and stained with annexin V-APC and PI in the dark for 20 min at room temperature. The stained cells were analyzed by flow cytometry (BD FACSCanto TM II). The percentages of apoptotic cells were measured using FlowJo software.

### 2.8 Measurement of mitochondrial membrane potential (MMP)

To measure the mitochondrial membrane potential (MMP), JC-1 assay kit (KeyGen Biotech, China) was used according to the manufacturer’s protocol. Control and treated cells were collected, stained with JC-1 dye, and incubated in the dark at 37°C for 30 min. The cells were immediately centrifuged, suspended in PBS, and analyzed using a flow cytometer. The ratio of JC-1 monomer fluorescence-expressing cells was used to quantify the loss of MMP.

### 2.9 Proteins extraction and western blot analysis

Following the indicated treatments, the cells and tissues were collected, washed once with PBS, and lysed with NP-40 lysis buffer (Beyotime, China), which contains protease and phosphatase inhibitors. The bicinchoninic acid (BCA) assay was used to measure protein concentrations, and equal amounts of lysates (40 μg) were run on 8%–15% sodium dodecyl sulfate polyacrylamide gels (SDS-PAGE). The proteins were transferred to polyvinylidene fluoride (PVDF) membranes and blocked with 5% skimmed milk/1× TBS/0.1% Tween for 2 h at room temperature. The membranes were incubated with primary antibodies at optimal dilutions overnight at 4°C, washed three times, and then incubated with the specific secondary antibodies conjugated with horseradish peroxidase (Millipore) for 2 h at room temperature. The blots were detected using an enhanced chemiluminescence detection solution and visualized on a ChemiDocTM imaging system. All Western blot results were repeated three or more times to ensure replicability.

### 2.10 Measurement of autophagic flux

HepG2 and Sk-Hep-1 cells were allowed to grow in 12-well plates to approximately 30% confluence. The cells were stably infected with StubRFP-SensGFP-LC3 lentivirus (Genechem, Shanghai, China). After the indicated treatments, the cells were washed twice with PBS, and then the nuclei were stained with Hoechst. A laser scanning confocal microscope (Carl Zeiss, Germany) was used to examine the cells. Autophagic flux was measured by quantifying GFP+/mRFP+ (yellow) and GFP−/mRFP+ (red) puncta using ImageJ software.

### 2.11 ELISA for SASP

The supernatants were collected from the culture of control and treated cells. The interleukin-6 (IL-6), interleukin-1 beta (IL-1β), and interleukin-1 alpha (IL-1α) were measured using enzyme-linked immunosorbent assay (ELISA) (Qiaodu Biotechnology, Shanghai, China) according to the manufacturer’s protocol.

### 2.12 Co-culture experiment

We hypothesized that senescent cancer cells promote the proliferation of cancer cells that do not have a senescence-like phenotype. To test this hypothesis, non-senescent (5 × 10^2^ cells/well) and senescent HepG2 or Sk-Hep-1 (3 × 10^3^ cells/well) were cultured alone or non-senescent cells were co-cultured with senescent cells or senescent cells pre-treated with TC in 12-well plates. Then, the medium was removed after 10 days, and the colonies were fixed for 30 min with methanol and stained for 20 min with 0.1% crystal violet. The colonies were observed using a light microscope.

### 2.13 Tumorigenesis assay of senescent cells

We hypothesized that senescent cancer cells promote tumor development. To test this hypothesis, we produced a nude mouse model with xenograft tumors using human HepG2 or Sk-Hep-1 cells. Nude mice were injected (100 µL vol) subcutaneously into the left upper flank with non-senescent HepG2 or Sk-Hep-1 cells, senescent HepG2 or Sk-Hep-1 cells into the right upper flank, non-senescent cells with senescent cells into the right lower flank, non-senescent cells with senescent cells pre-treated with TC into the left lower flank, respectively, and allowed to develop tumors. When the tumors reached about 50 mm^3^, tumor sizes of the test mice were measured every 3 days with a sliding caliper. The estimated tumor volumes were measured by the following equation: tumor volume= (a × b^2^)/2, where a is the length and b is the width in mm. After 3 weeks, mice were sacrificed, and the tumors were excised and photographed. All animal experiments were authorized by the Laboratory Animal Welfare and Ethics Committee of Fujian Medical University (grant number FJMU IACUC 2017-0093).

### 2.14 The senolytic efficiency of TC *in vivo*

Athymic nude mice (BALB/c-nu, 6–8 weeks of age; male, body weight: 20.0 ± 2.0 g) were purchased from Shanghai SLAC Laboratory Animal Co., Ltd. (Shanghai, China). To establish HCC xenograft model, mice were inoculated with 20 mm^3^ volume of HepG2 solid tumors into the right flanks using a trocar needle. When the tumors reached 100 mm^3^, the mice were then divided randomly into six groups of eight mice each and received different treatments: (a) control (0.9% saline, i. p.); (b) TC (100 mg/kg per day, i. p.); (c) TC (250 mg/kg per day, i. g); (d) ADR (6 mg/kg once a week, i. p.); (e) ADR and TC (100 mg/kg per day, i. p.); and (f) ADR and TC (250 mg/kg per day, i. g). To induce senescence *in vivo*, groups d, e, and f were treated with 6 mg/kg ADR intraperitoneally once a week for 2 weeks. Groups b, c, e, and f were subsequently treated with the indicated dosage of TC for 3 weeks, and the body weights of the mice and the tumor sizes were measured every 3 days to evaluate its antitumor and senloytic activities.

For cardiotoxicity analysis and blood tests, specific pathogen-free (SPF) male BALB/c mice (4–5 weeks old) were obtained from Guangdong Medical Experimental Animal Center (Foshan, China). Mice were randomized to either the control group or one of two treatment groups: the control group received vehicle (0.9% saline, i. p.), one treatment group received 6 mg/kg ADR (i.p.) once a week for 2 weeks, and 2 weeks after ADR treatment, the other treatment group received TC (100 mg/kg per day, i. p.) for 3 weeks.

At the end of the experiment, mice were sacrificed, and tumors and hearts were collected and fixed with 4% paraformaldehyde for immuno-histochemistry and pathological analysis. In addition, blood was collected by retro-orbital eye bleeding into anticoagulant tubes (Huabo Medical Equipment Co., Ltd., Heze, China) for routine blood and biochemical tests, and peripheral blood mononuclear cells (PBMCs) were isolated by Ficoll-Paque medium (Cytiva, Uppsala, Sweden) according to the manufacturer’s instructions. The lipophilic substrate 5-dodecanoylaminofluorescein di-β-D-galactopyranoside (C12FDG) was used to analyze the presence of SCs in PBMCs by flow cytometry. Frozen tissue sections were stained with 1x β-gal staining solution to quantify senescence. Apoptosis was quantified using the terminal deoxynucleotidyl transferase dUTP nick end labeling (TUNEL) protocol, and interleukins (IL)-6, IL-1β, and IL-1α were measured using ELISA in tumor tissues.

### 2.15 Statistical analysis

The values of triplicate experiments are presented as the mean ± standard deviation (SD). All data were analyzed by one-way or two-way analysis of variance (ANOVA) using GraphPad Prism 5 (San Diego, United States). *P < 0.05 was considered to be statistically significant.

## 3 Results

### 3.1 Characterization of TC

**FIGURE 1:**
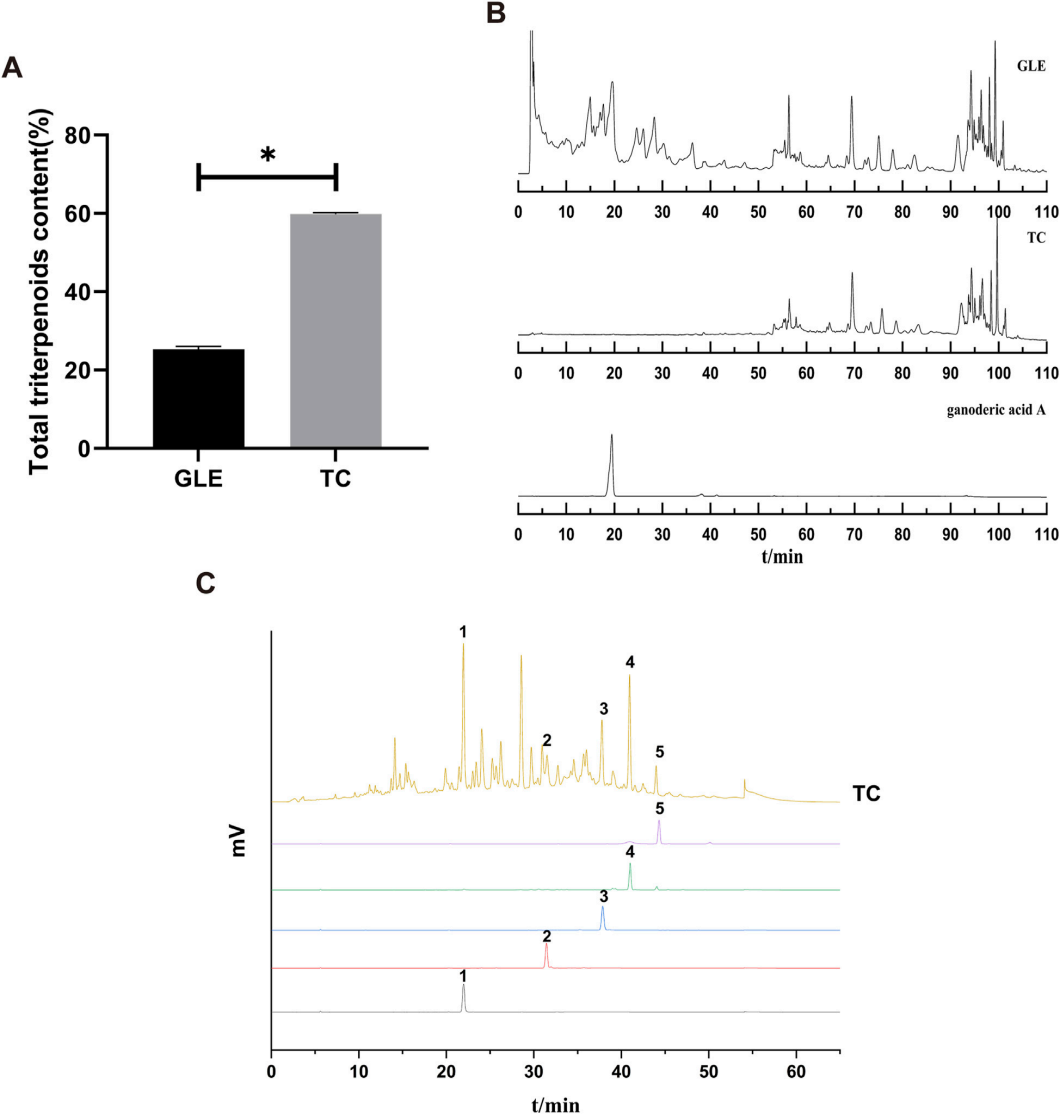
The characterization of TC. **(A)** The total triterpenes content of GLE and TC. Data were shown as mean ± SD (n = 3), *p < 0.05 *versus* GLE. **(B)** HPLC chromatograms of GLE, TC, and ganoderic acid A. **(C)** HPLC chromatograms of TC and reference substances. 1) ganodermanontriol, 2) ganodermanondiol, 3) ganoderiol B, 4) ganoderiol A, 5) ganoderal A.

TC was obtained from GLE with macroporous adsorption resin, and its yield was about 1% in the fruiting body of *G. lucidum*. The total triterpene content of TC was 60%, which was significantly higher than GLE (25.28%) (Figure 1A). HPLC chromatograms of GLE and TC showed that the retention time of chemical components in GLE was mainly distributed in two periods of 0–40 min and 50–90 min, while that of TC was mainly 50–90 min. Thus, it is clear that GLE treatment by macroporous adsorption resin eliminated the components with a retention time of 0–40 min containing ganoderic acid A, while TC with a retention time of 50–90 min was retained (Figure 1B). To characterize the triterpenes contained in TC, ganodermanondiol, ganodermanontriol, ganoderiol A, ganoderiol B, and ganoderal A were used as standard substances for HPLC analysis under the same conditions. The results showed that TC contained ganodermanontriol (21.94 min), ganodermanondiol (31.50 min), ganoderiol B (37.78 min), ganoderiol A (40.94 min) and ganoderal A (43.98 min) (Figure 1C). Taken together, these results indicate that TC is a triterpenoid complex from the fruiting body of G*. lucidum* with a definite chemical composition.

### 3.2 TC is a potential senolytic agent and selectively eliminates SCs *in vitro*

**FIGURE 2:**
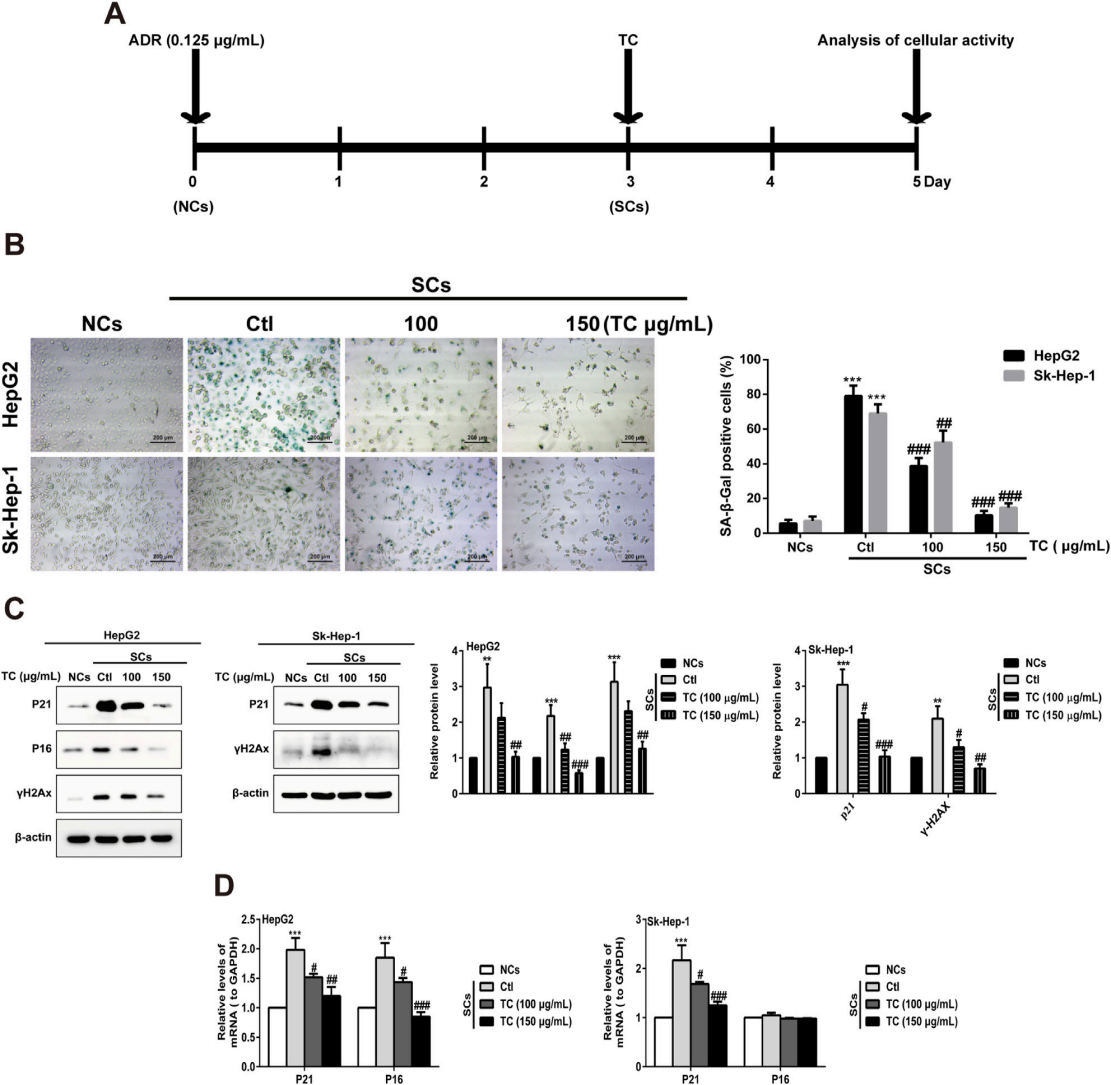
TC reduced SCs and downregulated senescence markers. **(A)** HCC cells were treated with ADR for 3 days and then TC for 2 days. **(B)** NCs, SCs and TC-treated SCs were stained with SA-β-Gal. **(C)** The levels of P21^Cip1/Waf1^, p16^Ink4a^, and γ-H2AX were determined by Western blotting in NCs, SCs, and TC-treated SCs. **(D)** P21^Cip1/Waf1^ and p16^Ink4a^ mRNA levels were determined by quantitative real-time PCR in NCs, SCs, and TC-treated SCs. Data were presented as mean ± SD (n = 3), **P < 0.01 and ***P < 0.001 *versus* NCs, ^#^P < 0.05, ^##^P < 0.01 and ^###^P < 0.001 *versus* Ctl.

While senescent cells are widely known to develop high activity of lysosomal SA-β-gal, we investigated the ability of TC to selectively eliminate senescent human HCC cells in a model of chemotherapy-induced senescence. Human HepG2 and Sk-Hep-1 cells were treated with 0.125 μg/mL ADR, which is slightly cytotoxic in HCC cells for 3 days to induce senescence (Lo Re et al., 2018). Cellular senescence induced by ADR was confirmed by increasing the levels of the cell cycle inhibitors p21^Cip1/Waf1^, p16^Ink4a^, and p53, as well as increased expression of the DNA damage marker γ-H2AX; however, p16^Ink4a^ protein was not expressed in Sk-Hep-1 cell line due to the heterogeneity of cell lines in response to senogenic agents (Supplementary Figure S1). SCs were subsequently treated with different concentrations of TC for 2 days, and senescence markers were analyzed (Figure 2A). To test the senolytic effect of TC, cell viability assays were performed on both NCs and SCs. As shown in Table 1, the IC50 values of TC at 48 h for NCs in HepG2 and Sk-Hep-1 cells were 175.54 μg/mL and 172.74 μg/mL, respectively, while for SCs, the IC50 values were 83.22 μg/mL and 81.55 μg/mL, respectively. Senolytic indices of TC for HepG2 and Sk-Hep-1 cells were 2.10 and 2.12, respectively. These results demonstrated that TC significantly reduced the viability of SCs more than NCs, indicating its ability to selectively eliminate SCs. We also compared the selectivity of TC against SCs to one of the well-known senolytic agents, the Bcl-2 family inhibitor navitoclax (ABT-263) (Zhu et al., 2016). It was found that SCs were significantly more sensitive than NCs to navitoclax treatment. Correspondingly, the same selective effect was detected following TC treatment of NCs and SCs (Supplementary Figure S2). In addition, SA-β-gal activity was determined by the colorimetric substrate X-gal (5-bromo-4-chloro-3-indolyl-β-D-galactopyranoside) to measure senescence. A light microscope was used to count X-gal-positive cells. After treatment with TC, the number and density of X-gal-positive cells were significantly decreased. The SA-β-gal staining in SCs was visualized using a quantitative graph (Figure 2B). To further confirm the obtained results, we measured the protein expression of molecular senescence markers by Western blotting. The results showed that p21^Cip1/Waf1^, P16^INK4a^, and γ-H2AX proteins were significantly decreased in SCs after TC treatment (Figure 2C). Besides, the gene expression of P21^Cip1/Waf1^ and p16^Ink4a^ in SCs measured by qPCR after TC treatment were similar to their proteins (Figure 2D). Taken together, these findings suggest that TC has a potential senolytic effect on ADR-induced senescent HCC cells.

**TABLE 1:** IC50 values of TC against NCs and SCs in HepG2 and Sk-Hep-1 cells.

Cell lines	IC50 (μg/mL)		Senolytic index IC50 ratio (NCs/SCs)
	NCs	SCs	
HepG2	175.54	83.22	2.10
Sk-Hep-1	172.74	81.55	2.12

### 3.3 TC induces caspase-dependent apoptosis of senescent cells through the mitochondrial pathway

**FIGURE 3:**
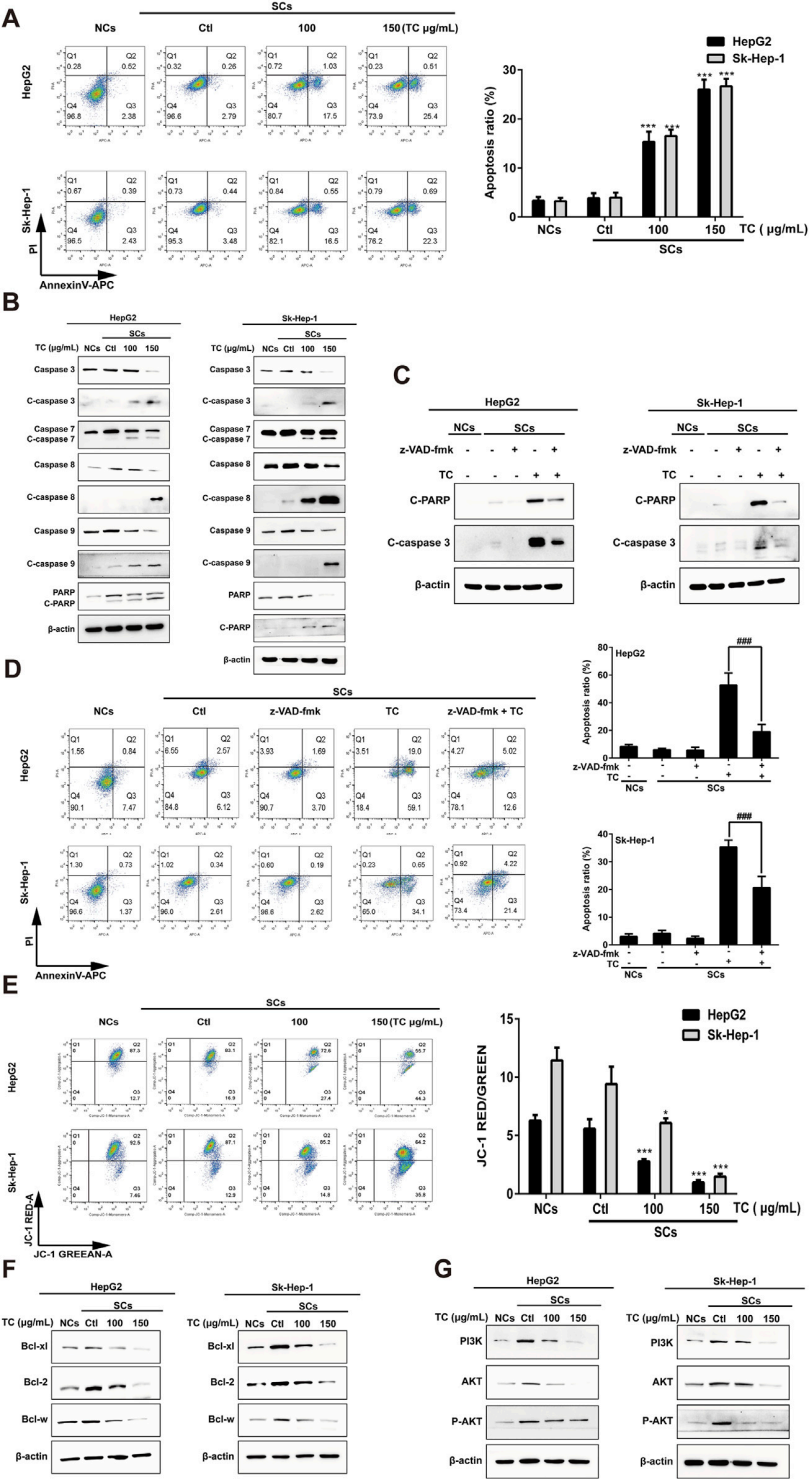
TC-induced apoptosis in SCs. **(A)** The percentage of apoptotic cells was analyzed by flow cytometry in NCs, SCs, and TC-treated SCs. **(B)** The expression levels of apoptosis-related proteins were determined by Western blotting in NCs, SCs, and TC-treated SCs. **(C)** The expressions of cleaved PARP and cleaved caspase-3 in SCs pre-treated with z-VAD-fmk were detected by Western blotting. **(D)** Quantification of the apoptotic ratio (annexin V/PI staining) in SCs pre-treated with z-VAD-fmk by flow cytometry. **(E)** The mitochondrial membrane potential was determined by flow cytometry in NCs, SCs, and TC-treated SCs. **(F)** The expression levels of Bcl-xl, Bcl-2, and Bcl-w proteins were determined by Western blotting in NCs, SCs, and TC-treated SCs. **(G)** The expression levels of PI3K, Akt, and P-Akt were determined by Western blotting in NCs, SCs, and TC-treated SCs. Data were presented as the mean ± SD (n = 3), *P < 0.05 and ***P < 0.001 *versus* Ctl, ^###^P < 0.001 *versus* TC treatment.

Senescent tumor cells have upregulated anti-apoptotic and pro-survival molecular pathways, such as Bcl-2 protein family and PI3K/Akt signaling pathway that reinforce resistance to apoptosis (Zhu et al., 2015). SCs induced by ADR were utilized to determine the impact of TC on apoptosis using annexin V–APC/PI staining. It was found that TC induced apoptosis of SCs in a dose-dependent manner (Figure 3A). To analyze the molecular mechanism behind TC-induced apoptosis in SCs, we determined the molecular changes associated with apoptosis. It was reported that caspases are the central signaling proteins that play an essential role in different forms of apoptosis (Utz and Anderson, 2000). Western blot analysis showed that TC increased the cleavage of caspase-3,-7,-8, and -9, and PARP in a dose-dependent manner in SCs (Figure 3B; Supplementary Figure S3). To confirm the importance of caspase stimulation in TC-induced apoptosis, SCs were pre-treated with the general caspase inhibitor z-VAD-fmk. The cleavage of PARP and caspase-3 induced by TC (150 μg/mL) was dramatically inhibited by z-VAD-fmk (Figure 3C; Supplementary Figure S4). Besides, TC-induced apoptosis of SCs was accordingly reduced by z-VAD-fmk (Figure 3D).

Next, we investigated whether the mitochondrial function in SCs could be affected by TC. We tested disruption in mitochondrial membrane potential (MMP) using JC-1 staining. After TC treatment for 48 h, MMP of SCs was significantly decreased in a dose-dependent manner, which can be demonstrated by an increase in JC-1 fluorescence (Figure 3E). These findings suggest that TC triggers mitochondrial dysfunction, resulting in MMP reduction, enhancing mitochondrial permeability, and then activation of caspase-dependent and mitochondrial-mediated pathways of apoptosis in SCs.

It was previously reported an upregulation of the Bcl-2 family members (Bcl-xl, Bcl-2, and Bcl-w) during senescence, which account for the resistance of senescent cells to apoptosis (Baar et al., 2017). To investigate the mechanism by which TC selectively triggers the elimination of ADR-induced SCs, we determined the effect of TC on the expression levels of these proteins by Western blotting. It was found that the expression levels of the anti-apoptotic proteins (Bcl-xl, Bcl-2, and Bcl-w) were significantly increased in ADR-induced SCs, and the expression levels of these proteins were significantly decreased by TC treatment in a dose-dependent manner in SCs (Figure 3F; Supplementary Figure S5), which was similar to the action of navitoclax (Supplementary Figure S6). These findings suggest that the anti-apoptotic Bcl-2 family members confer the resistance of ADR-induced SCs to apoptosis, and their inhibition by TC leads to selective elimination of these cells via apoptosis induction. In addition, TC treatment for 48 h was enough to downregulate the levels of PI3K, AKT, and its active phosphorylated form, contributing to the induction of apoptosis in SCs (Figure 3G; Supplementary Figure S7).

### 3.4 TC inhibits the late stage autophagy in senescent cells

**FIGURE 4:**
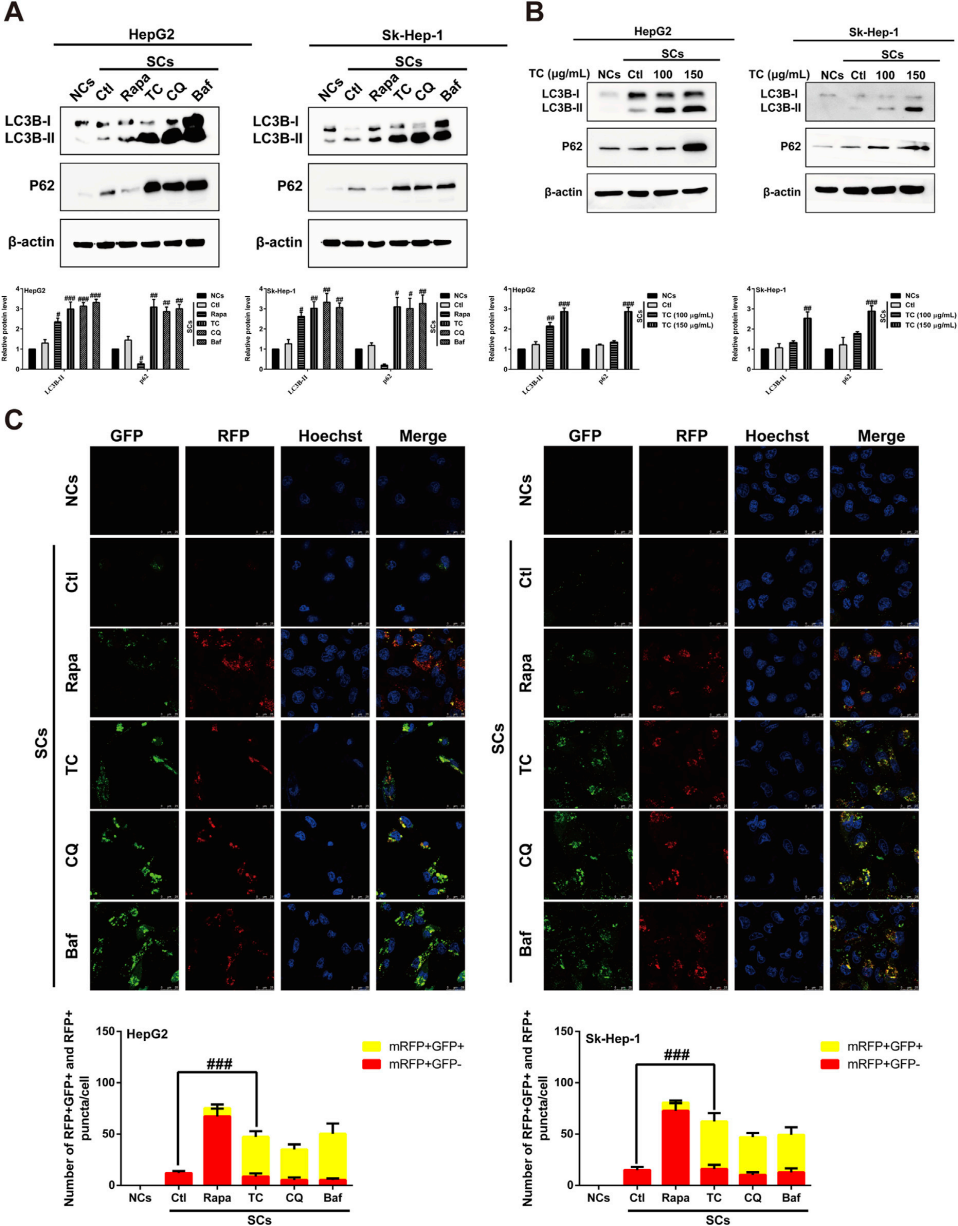
TC inhibited autophagy flux in SCs. **(A)** Western blotting was used to determine the expression levels of LC3BI/II and P62 in NCs, SCs, and Rapa, TC, CQ, and Baf-treated SCs. **(B)** Western blotting was used to measure the expression levels of autophagy-related proteins in NCs, SCs, and TC-treated SCs. **(C)** Confocal microscopic analysis of red and yellow puncta in NCs, SCs, and Rapa, TC, CQ and Baf-treated SCs. The bar charts show the quantification of red and yellow puncta per cell. Data were presented as the mean ± SD (n = 3), ^#^ *p* < 0.05, ^##^ *p* < 0.01 and ^###^ *p* < 0.001 *versus* Ctl.

It has been reported that induction of cellular senescence by ADR is associated with activation of autophagy (Di et al., 2009; Yang et al., 2012). To study the effect of TC on the modulation of autophagy in SCs, we first observed the effect of TC on the specific autophagy biomarker microtubule-associated protein 1A/1B-light chain 3 (LC3) using Western blotting. TC significantly increased LC3B-II levels in SCs, which is a critical sign of autophagy initiation (Figure 4A). However, the upregulation in the LC3B-II level triggered by TC in SCs may be associated with either increased autophagosome formation due to increased autophagic activity or decreased autophagosome degradation due to the inhibition of autophagy at the late stage (Ragazzoni et al., 2013). To distinguish between these two possibilities, we compared the effect of TC to the autophagy inducer rapamycin (Rapa), and the late stage autophagy inhibitors chloroquine (CQ) and bafilomycin A1 (Baf) on the expression of LC3B-II and p62. It was found that Rapa increased the expression of LC3B-II, and reduced that of p62. In contrast, TC, along with CQ and Baf increased the expression of both LC3B-II and p62 (Figure 4A). Additionally, TC increased the expression of both LC3B-II and p62 in a concentration-dependent manner in SCs (Figure 4B), similar to the effects of CQ and Baf. It has been reported that p62 is implicated in the formation of autophagosomes and constitutively degraded through the autophagic pathway by specific binding to LC3 at the late stage of autophagy. Therefore, the accumulation of p62 can be considered as a marker for autophagy inhibition at the late stage (Pankiv et al., 2007; Mathew et al., 2009). Thus, our data indicate that TC inhibits autophagy flux in SCs at the late stage of autophagy.

To confirm the inhibition of autophagic flux by TC treatment in SCs, autophagy flux was measured by transfecting SCs with a tandem fluorescent-tagged LC3 reporter plasmid (GFP–mRFP–LC3) (Kimura et al., 2007). The probe GFP–mRFP–LC3 emits both green and red fluorescence at the neutral pH of autophagosomes, and the merging of both results in yellow fluorescence (mRFP+ GFP+), indicating that the autophagosome does not fuse with the lysosome, implying impaired autophagy. At the late stage of autophagy, autophagosomes fuse with lysosomes, resulting in a low intra-lysosomal pH that facilitates proteolysis. In these acidic compartments, the green fluorescence of GFP is quenched, leaving only the red fluorescence of mRFP (mRFP+ GFP-), which indicates normal autolysosome maturation. Red (mRFP+ GFP-) and yellow (mRFP+ GFP+) puncta per cell were quantified to assess autophagic flux. It was found that TC-treated SCs exhibited an accumulation of yellow puncta, consistent with the effects of CQ and Baf. However, the number of red puncta per cell increased in the presence of Rapa (Figure 4C). These findings suggest that TC reduces autophagy flux and blocks the late stage of autophagy in SCs.

Finally, we investigated whether blocking autophagy could enhance TC-induced apoptosis in SCs. It was found that ADR-induced senescence could activate autophagy (Figure 4C) and exhibit anti-apoptosis properties, as the apoptosis rate was very low, similar to NCs (Supplementary Figure S8A). Treatment of SCs with TC or CQ, which blocked autophagy (Figures 4A, C), significantly increased the apoptosis rate in SCs, while combining TC and CQ further enhanced apoptosis in SCs (Supplementary Figure S8A). In addition, the combination of TC and CQ increased the cleavage of PARP and caspase-3 proteins compared to TC treatment alone (Supplementary Figure S8B). These findings indicate that ADR-induced senescence is accompanied by protective autophagy, and blocking autophagy with TC improves the sensitivity of SCs to apoptosis.

### 3.5 TC attenuates the SASP and suppresses the growth stimulation induced by senescent cells *in vitro* and *in vivo*

**FIGURE 5:**
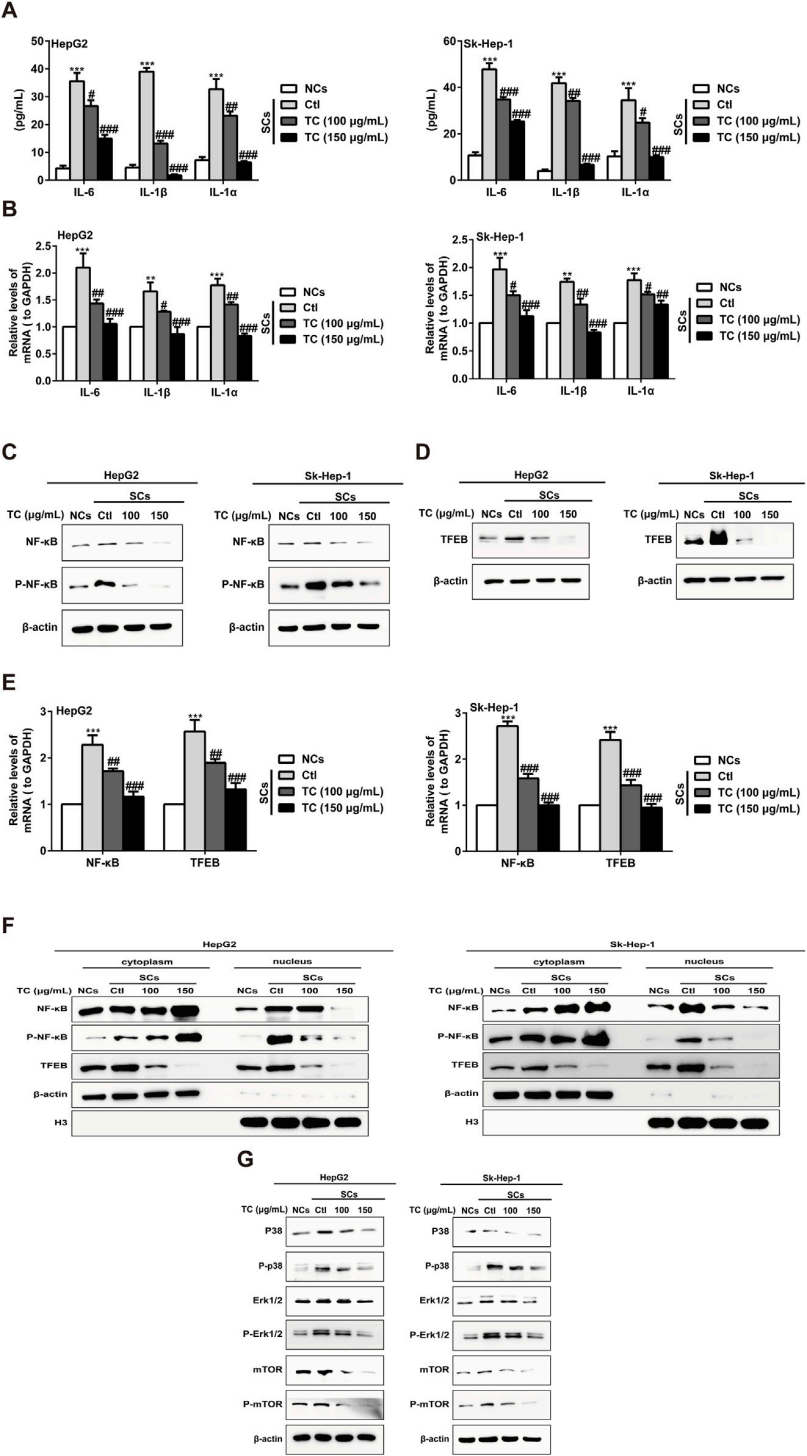
TC inhibited the SASP in SCs. **(A)** IL-6, IL-1β, and IL-1α were analyzed by ELISA in NCs, SCs, and TC-treated SCs. **(B)** IL-6, IL-1β, and IL-1α mRNA levels were measured by quantitative real-time PCR in NCs, SCs, and TC-treated SCs. **(C, D)** The levels of NF-κB, P-NF-κB, and TFEB were measured by Western blotting in NCs, SCs, and TC-treated SCs. **(E)** NF-κB and TFEB mRNA levels were measured by quantitative real-time PCR in NCs, SCs, and TC-treated SCs. **(F)** Western blotting was used to measure the cytoplasmic and nuclear fraction of NF-κB, P-NF-κB, and TFEB in NCs, SCs, and TC-treated SCs. **(G)** The levels of P38, P-p38, ERK, P-ERK, mTOR, and P-mTOR were measured by Western blotting in NCs, SCs, and TC-treated SCs. Data were presented as the mean ± SD (n = 3), **P < 0.01 and ***P < 0.001 *versus* NCs, ^#^ *P* < 0.05, ^##^ *P* < 0.01 and ^###^ *P* < 0.001 *versus* Ctl.

To investigate the capacity of TC to modulate the SASP in SCs, we examined the effect of TC treatment on the production of major SASP components, as well as the pro-proliferative activity of SCs *in vitro* and *in vivo* (Acosta et al., 2013). The increased SASP components in SCs, including IL-6, IL-1β, and IL-1α were markedly reduced after TC treatment (Figure 5A). Correspondingly, TC treatment significantly reduced the gene expression of these components, as evidenced by qPCR (Figure 5B). The SASP is regulated by transcription factors and their signaling pathways, including NF-κB, TFEB, Mitogen-activated protein kinases (MAPKs), which are mediated by ERK, c-Jun amino-terminal kinases (JNK), and p38MAPK, and the PI3K-AKT-mTOR pathways (Sun et al., 2018). To investigate the molecular mechanism by which TC induces inhibition of the SASP, we observed the effect of TC on these signaling pathways. The results demonstrated that TC treatment decreased NF-κB protein expression and its phosphorylation (Figure 5C; Supplementary Figure S9A) alongside the expression of TFEB protein (Figure 5D; Supplementary Figure S9B). Similarly, the gene expression of NF-κB and TFEB was significantly reduced by TC treatment in SCs (Figure 5E). Furthermore, it was found that TC treatment in SCs suppressed the levels of NF-κB and P-NF-κB in the nucleus, causing them to accumulate in the cytoplasm, indicating that TC may decline the translocation of NF-κB protein from the cytoplasm to the nucleus. Meanwhile, TFEB levels were significantly reduced in the cytoplasm and nucleus following TC treatment in SCs (Figure 5F; Supplementary Figure S10). Moreover, TC treatment was found to significantly reduce the levels of P38, P-p38, ERK, P-ERK, mTOR, and P-mTOR in SCs (Figure 5G; Supplementary Figure S11). These findings suggest that TC inhibits the SASP in SCs through the inhibition of NF-κB, TFEB, P38, ERK, and mTOR signaling pathways.

It was reported that different SASP-derived substances have the ability to promote the growth and recurrence of cancer (Zhang et al., 2021). Therefore, we tested whether ADR-induced SCs could promote the growth of NCs through the SASP, and whether TC could inhibit this growth stimulation via its senolytic effect and suppression of the SASP *in vitro* and *in vivo*. The effect of SCs on NCs growth was examined *in vitro* using the colony formation assay. It was found that SCs alone had no colony-forming ability and the colony numbers of NCs co-cultured with SCs were markedly higher compared to NCs alone. By contrast, the growth stimulation caused by SCs was declined by TC pre-treatment of SCs and co-cultured with NCs, as demonstrated by the reduction in the colony numbers (Supplementary Figure S12A). These data suggest that TC inhibits SCs-induced hyperproliferation *in vitro*. Next, *in vitro* findings were confirmed using human HCC xenografts in nude mice. It was found that SCs alone did not form tumors. However, when SCs were co-injected with NCs, the tumor volumes developed in animals significantly increased compared to NCs alone. Moreover, the malignant promotion ability of SCs was attenuated when SCs were pre-treated with TC, as confirmed by reduced tumor volumes when the pre-treated SCs were co-injected with NCs (Supplementary Figure S12B). These data indicate that TC treatment may suppress SCs-induced tumor growth promotion *in vivo*, possibly through the elimination of SCs or the suppression of SASP, and further studies are needed to confirm the underlying mechanism.

### 3.6 The sequential administration of ADR and TC enhances the antitumor effect *in vivo*

**FIGURE 6:**
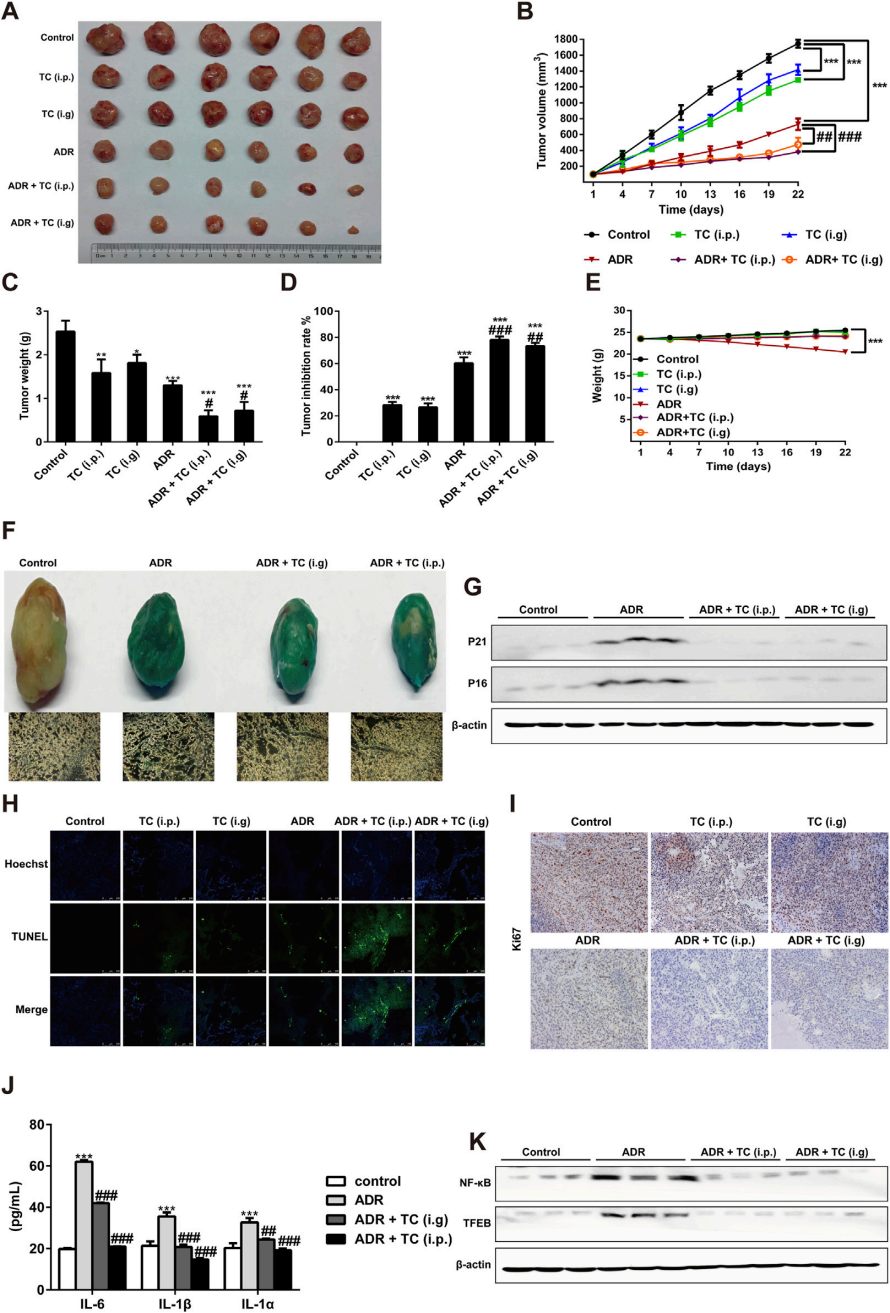
TC enhanced the antitumor effect of ADR *in vivo*. **(A)** Tumor sizes in treatment groups. **(B)** Tumor volumes in treatment groups were measured. **(C, D)** The tumor weights and tumor inhibition rate were calculated in treatment groups. **(E)** The body weights of animals in treatment groups were measured. **(F)** SA-β-Gal staining in tumors and frozen sections from HepG2 xenograft treated with vehicle, ADR and TC. **(G)** Western blotting was used to measure the expression levels of P21^Cip1/Waf1^ and p16^Ink4a^ in tumors treated with vehicle, ADR, and TC. **(H)** The TUNEL assay was performed on frozen sections of tumors from treatment groups. **(I)** Frozen sections of tumors from treatment groups were stained for Ki67. **(J)** The levels of IL-6, IL-1β, and IL-1α were measured by ELISA in tumors treated with vehicle, ADR, and TC. **(K)** Western blotting was used to measure the expression levels of NF-κB and TFEB in tumors treated with vehicle, ADR, and TC. Data were presented as the mean ± SD, *p < 0.05, **P < 0.01 and ***P < 0.001 *versus* the control, ^#^ *P* < 0.05, ^##^ *P* < 0.01 and ^###^ *P* < 0.001 *versus* ADR treatment.

The National Cancer Institute Workshop on Radiation, Senescence and Cancer introduced the concept of “one-two punch” cancer therapy. This approach involves using a chemotherapeutic agent to kill proliferative tumor cells and induce tumor cell senescence followed by the selective elimination of senescent cells with senolytics to enhance treatment outcomes for cancer patients (Prasanna et al., 2021). Here, we designed a “one-two punch” regimen (Supplementary Table S1) to determine whether TC could clear ADR-induced SCs in HCC xenograft model to enhance the antitumor efficacy of ADR. After tumor growth to 100 mm^3^, the tumor-bearing mice were treated with 6 mg/kg ADR by intraperitoneal injection once a week for 2 weeks to induce senescence, which was demonstrated by enhancing the activity of SA-β-gal and reducing Ki67 levels in tumor sections (Supplementary Figures S13A, B). After 2 weeks of ADR treatment to induce senescence in tumor-bearing mice, TC was given orally or intraperitoneally for 3 weeks. The results showed that the sequential combination of ADR with TC (i.p. or i. g) significantly inhibited tumor growth compared to groups administered each individual treatment (Figures 6A, B). Tumor weights were the lowest in the groups that received the combination treatment (Figure 6C). At the end of the treatment, the tumor growth inhibition rates in groups of TC (i.p.), TC (i.g), ADR, ADR + TC (i.p.), and ADR + TC (i.g) were 28.2% ± 2.5, 26.5% ± 3.0, 60.3% ± 4.4, 78.2% ± 2.6% and 73.4% ± 2.5, respectively (Figure 6D). These data suggest that the sequential combination of ADR and TC (i.p. or i. g) significantly reduces the growth of HCC *in vivo* compared to single treatments.

Compared to the control group, the body weights of animals treated with TC (i.p. or i. g) did not show significant change during the treatment, while the body weights of animals treated with ADR were significantly reduced. Interestingly, the reduction in the body weights of animals treated with ADR was significantly recovered after TC treatment (Figure 6E). Next, we investigated the effect of TC on ADR-induced senescence in HCC tumor samples. Consistent with our *in vitro* results, the combination-treated groups demonstrated a significant reduction in SA-β-gal activity and the levels of p21^Cip1/Waf1^ and p16^Ink4a^ proteins compared to ADR-treated tumors (Figures 6F, G; Supplementary Figure S14A), indicating that TC treatment effectively reduced senescence in tumor tissues. The results of TUNEL assay revealed that the combination-treated groups had a higher TUNEL signal compared to monotherapy groups, indicating an increased number of apoptotic SCs in tumor tissues and enhancing the antitumor activity (Figure 6H). In addition, immunohistochemistry of tumor tissues showed that the combination treatment resulted in lower levels of Ki67 expression, a proliferation marker, compared to the single treatment groups (Figure 6I). Finally, analysis of common SASP markers in tumor tissues demonstrated that ADR markedly stimulated the expression of IL-6, IL-1β, and IL-1α, while TC significantly inhibited their expressions (Figure 6J). Also, TC treatment markedly decreased the protein levels of NF-κB and TFEB in tumor tissues compared to the ADR-treated group (Figure 6K; Supplementary Figure S14B). These findings suggest that the sequential combination of chemotherapy with TC can eliminate chemotherapy-induced senescence and enhance the anti-tumor efficacy, providing preclinical evidence of TC as an adjuvant therapy for liver cancer.

### 3.7 TC restores ADR-induced cardio, hematological, and biochemical toxicities

It is well known that the administration of ADR, as a chemotherapy drug results in serious toxicities, including cardiac, hematological, hepatic, and renal toxicities (Piegari et al., 2013; Wang et al., 2013). We observed that the body weights of mice in the ADR treatment group decreased significantly, while those in the group treated with the combination of ADR and TC nearly returned to normal. Therefore, we are interested in exploring whether the reduction in ADR-induced toxicity by TC is associated with the elimination of senescent cells.

**FIGURE 7:**
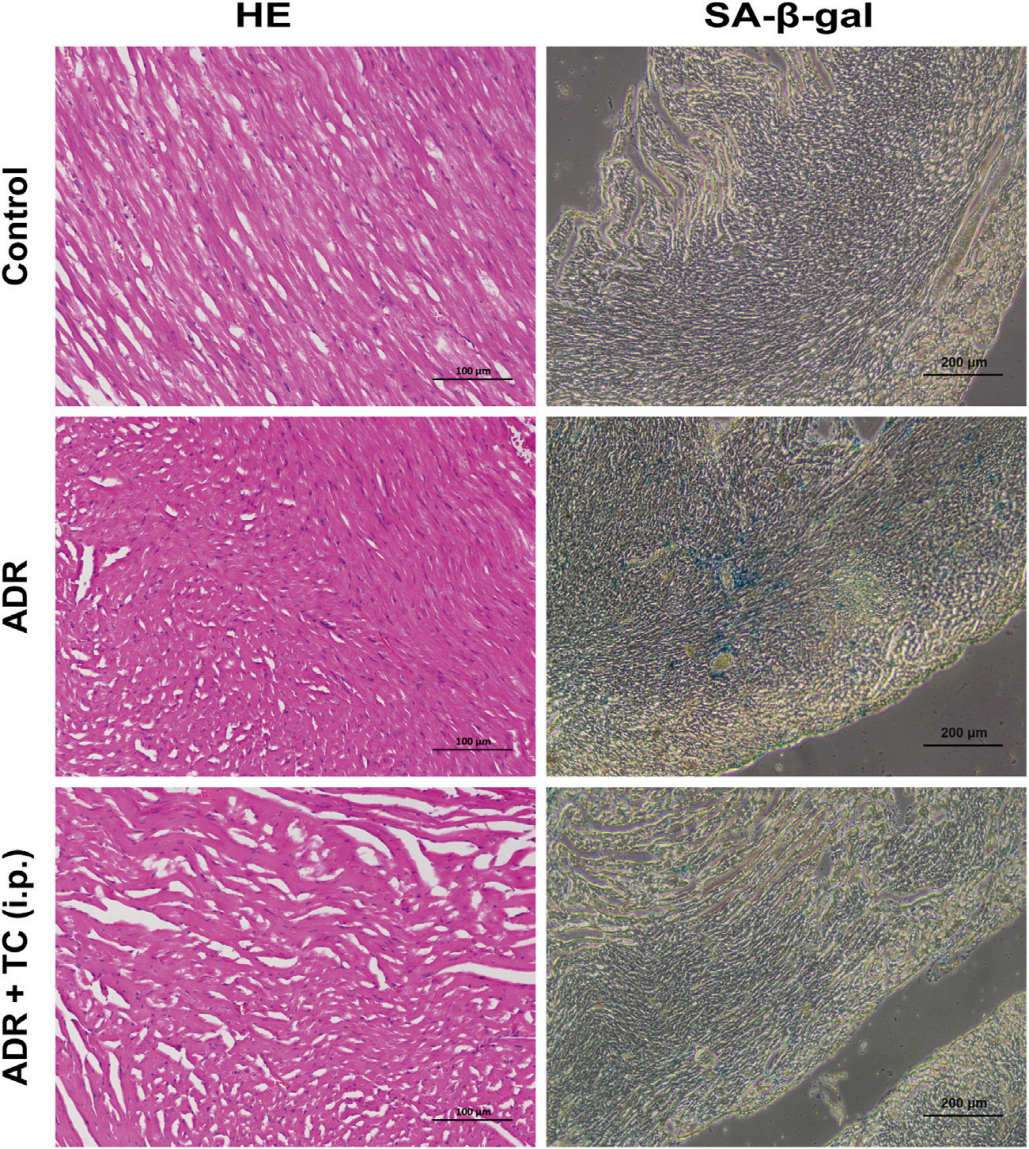
Representative images of HE and SA-β-gal staining in different treatment groups.

HE staining was used to observe pathological changes in the cardiac tissues of mice treated with ADR or the sequential combination of ADR and TC. It was found that the ADR group showed fiber breakage in the cardiac tissues. However, TC treatment partially reversed the damage of the cardiac tissues. Next, we investigated whether ADR could induce senescence in cardiac tissues, and whether TC treatment could reduce cardiac senescence in this animal model. The results showed that SA-β-gal activity in the cardiac tissues was higher in the ADR group compared to the control, while the combination-treated group demonstrated a significant reduction in SA-β-gal activity compared to the ADR group (Figure 7). These results suggest that the cardiac damage caused by ADR treatment may be associated with its induction of senescence in myocardial cells, whereas TC may alleviate ADR-induced cardiotoxicity by reducing senescence. After that, we determined the number of SCs in PBMCs following ADR treatment. The results revealed that the accumulation of SCs in the ADR group was significantly higher than in the control group, with the highest levels detected after 2 weeks of ADR treatment (Supplementary Figure S15).

The hematological and biochemical profiles in different treatment groups are shown in Table 2. There were no statistically significant differences in red blood cells and hemoglobin levels between the control and treatment groups. However, the number of white blood cells, lymphocytes, monocytes, granulocytes, and platelets were significantly decreased in the ADR group compared to the control group. Notably, these reductions were recovered in the combination-treated group. In terms of liver and kidney functions, ADR triggered a significant reduction in aspartate aminotransferase, alanine transaminase, urea, and creatinine levels compared to the control group. These levels were reversed towards normal in the combination-treated group. These findings indicate that the elimination of senescent cells by TC may be a promising strategy to overcome ADR-associated toxicities and its clinical limitations.

**TABLE 2:** The effect of ADR and TC on routine blood and biochemical tests (mean ± SD).

Items	Treatment groups		
	Control	ADR	ADR + TC
Blood routine			
RBC (X10^12^/L)	10.3 ± 0.75	11.0 ± 0.24	10.87 ± 0.15
HGB (g/L)	179.0 ± 0.51	180.0 ± 0.03	178.0 ± 0.02
WBC (X10^9^/L)	6.9 ± 0.15	3.5 ± 0.20***	5.5 ± 0.16^###^
Lymph (X10^9^/L)	5.2 ± 0.15	3.2 ± 0.07***	4.9 ± 0.02^###^
Mon (X10^9^/L)	0.2 ± 0.02	0.1 ± 0.001***	0.17 ± 0.01^###^
Gran (X10^9^/L)	1.6 ± 0.07	0.8 ± 0.02***	1.1 ± 0.05^###^
PLT (X10^9^/L)	1018.0 ± 0.58	752.0 ± 1.19***	930.0 ± 1.28^###^
Liver functions			
AST (U/L)	18.1 ± 1.5	35.2 ± 1.5***	25.9 ± 2.1^##^
ALT (U/L)	44.2 ± 1.2	78.2 ± 3.5***	65.3 ± 1.5^#^
Kidney functions			
Urea (mmol/L)	20.3 ± 1.5	33.8 ± 2.2***	26.3 ± 1.5^##^
Cr (μmol/L)	19.7 ± 1.3	68.2 ± 1.5***	45.7 ± 3.5^##^

Control: n = 8, ADR and ADR + TC: n = 6, ^***^ *P* < 0.001 *versus* control, ^#^ *P* < 0.05, ^##^ *P* < 0.01 and ^###^ *P* < 0.001 *versus* ADR treatment. RBC: red blood cell; HGB: hemoglobin; WBC: white blood cell; Lymph: lymphocyte; Mon: monocyte; Gran: granulocyte; PLT: platelet; AST: aspartate aminotransferase; ALT: alanine transaminase; Cr: creatinine.

## 4 Discussion

Hepatocellular carcinoma is the third leading cause of cancer-related deaths worldwide and one of the most prevalent malignancy in China. Due to the deficiency of effective targeted drugs for HCC, it is critical to find more suitable approaches to reduce the occurrence and development of HCC (Kensler et al., 2003).

Cellular senescence is a physiological process in which irreversible cell cycle arrest occurs and the cells lose their ability to differentiate but continue to be metabolically active for an extended period of time (Evan and d'Adda di Fagagna, 2009). Senescence can be mediated via various mechanisms, including telomere shortening (replicative senescence), activation of oncogenes, such as Ras (oncogene-induced senescence), and DNA damage (premature, accelerated, or stress-induced senescence) (Gewirtz et al., 2008). Recent studies have demonstrated that cytotoxic drugs, such as adriamycin and camptothecin induce terminal growth arrest with senescence-like features in tumors. P53 and the cyclin-dependent kinase (CDK) inhibitor p21^Cip1/Waf1^ are important players in this mechanism (Chang et al., 2002; Han et al., 2002). Although cellular senescence is known to elicit antitumor responses, it also leads to the production of various mediators by senescent cells, such as pro-inflammatory cytokines, chemokines, growth factors, and proteases. These mediators have an impact on neighboring non-senescent cells and their microenvironment through autocrine and paracrine effects. Therefore, this process contributes to the recurrence and metastasis of tumors (Van Deursen, 2014).

It is well known that senescent cells depend on pro-survival and anti-apoptotic pathways to increase their resistance to cell death. These pathways include the Bcl-2 family proteins (Bcl-xl, Bcl-2, and Bcl-w), PI3K/AKT, hypoxia-inducible factor (HIF-1α), Serpins (PAI-1 and PAI-2), Hsp90, and autophagy (Sikora et al., 2019). Hence, many efforts have been directed to the development of senolytics for cancer therapy, especially when chemotherapy-induced senescent cells is combined with senolytics that selectively clear senescent cancer cells, which is known as “one-two punch” strategy (Wang C. et al., 2019). The current study experimentally verified the effectiveness of combining chemotherapy-induced senescent HCC cells with senolytic agents in liver cancer.

To date, natural compounds have been identified as effective senolytic agents because of their safety and low toxicity (Zhu M. et al., 2020). Various studies have shown the role of *G. lucidum* in treating and preventing senescence-related diseases (Sudheesh et al., 2009; Sudheesh et al., 2010). However, the senolytic effect of *G. lucidum* against senescent cancer cells has not yet been reported.

In this work, ADR treatment resulted in SCs, which was confirmed using various senescence markers, such as SA-β-Gal activity, the cell cycle inhibitors p21^Cip1/Waf1^, p16^Ink4a^, and p53, the DNA damage marker γ-H2AX, autophagy activation, and the release of SASP factors as IL-6, IL-1β, and IL-1α. Numerous studies have demonstrated that senescent cells evade apoptosis by increasing the expression of the anti-apoptotic Bcl-2 family proteins (Chang et al., 2016). These proteins protect senescent cells against both intrinsic (mitochondrial pathway) and extrinsic (death receptor pathway) pro-apoptotic signals by binding to Bax/Bak, thereby disrupting the release of mitochondrial cytochrome *c*, and caspase signaling activation. This allows senescent cells to survive and induce a variety of biological processes (Czabotar et al., 2014; Correia et al., 2015). In this work, TC treatment significantly downregulated Bcl-xl, Bcl-2, and Bcl-w, and reduced MMP resulted in the activation of caspase-3, caspase-7, caspase-8 and caspase-9, and cleaved PARP in SCs. These results indicate that TC triggers apoptosis via both the extrinsic and intrinsic pathways, and ultimately eliminates SCs. Additionally, the downregulation of pro-survival PI3K, AKT, and its active phosphorylated form also contributes to TC-induced apoptosis in SCs.

Autophagy is a catabolic mechanism in which organelles, such as mitochondria and lysosomes, and proteins are degraded by intracellular degradation system to provide metabolites and sustain the cellular energy supply (Glick et al., 2010). Autophagy can support tumor cell survival under cellular stresses, including starvation, hypoxia, and some types of therapy, such as chemotherapy and radiotherapy (Yue et al., 2003; Thorburn et al., 2014). It was reported that inhibition of autophagy in HCC cells sensitized the cells to the targeted therapy (Pan et al., 2014). While senescence and autophagy are commonly thought to be two separate cellular responses, there is accumulating evidence that both of them are functionally linked in response to various forms of stress (Huang et al., 2014). Furthermore, previous studies have demonstrated that autophagy also plays a critical role in the SASP production by activating the protein synthesis of key cytokines, including IL-6 and IL-8 (Narita et al., 2011). It was reported that blocking autophagy induced by therapy-mediated senescent cancer cells resulted in senescent cell death (Dörr et al., 2013). In clinical trials, the autophagy inhibitor CQ and its derivative hydroxychloroquine (HCQ) have shown signs of efficacy for cancer treatment (Amaravadi et al., 2011). In this study, ADR treatment induced both senescence and autophagy in HCC cells, but the cells did not die, indicating the cytoprotective role of autophagy in SCs. Additionally, blocking autophagy by TC treatment triggered apoptosis in ADR-induced SCs. Thus, inhibiting autophagy is a promising strategy for alleviating senescence induced by chemotherapy in HCC cells.

Another significant hallmark of senescence is the acquisition of the SASP, which is uniformly activated in cancer cells in response to radiation or chemotherapy (Rodier et al., 2009). The paracrine effects of the SASP on neighboring tumor cells and their microenvironment are mainly responsible for some of the pro-tumorigenic outcomes associated with senescent cells (Sagiv and Krizhanovsky, 2013; Hernandez-Segura et al., 2018). Multiple signaling pathways, such as NF-κB, TFEB, MAPK, and the PI3K-AKT-mTOR pathways are involved in the SASP regulation (Malaquin et al., 2016). NF-κB signaling is the major pathway that activates the SASP by increasing the transcription of many SASP components (Chien et al., 2011). Transcription factor TFEB has recently been identified as a regulator of IL-6 expression, lysosomal biogenesis, and autophagy (Wang S. et al., 2019). MAPK pathway is activated in senescent cells and affects the SASP through increasing the transcription activity of NF-κB (Freund et al., 2011). In addition, mTOR triggers the SASP production by controlling the translation of specific mRNAs included in the regulation of the SASP (Laberge et al., 2015). Recently, the SASP modulators mainly target these signaling pathways to block the SASP activity in senescent cells (Moiseeva et al., 2013). In this work, we investigated that TC markedly inhibited well-defined SASP components, such as IL-6, IL-1β, and IL-1α in SCs by inhibiting NF-κB, TFEB, P38, ERK, and mTOR signaling pathways. In addition, TC effectively reduced the number of SCs by inducing apoptosis, which also contributed to a reduction in the SASP.

To investigate the senolytic activity of TC *in vivo*, we designed a “one-two punch” therapy scheme. The senescence-inducer ADR was administered alone to tumor-bearing mice once a week for 2 weeks as the first punch. ADR not only killed tumor cells but also induced senescence in tumor cells. After 2 weeks, a large number of senescent cells were found in the tumor tissues, which was confirmed by high levels of SA-β-gal activity, p21^Cip1/Waf1^and p16^Ink4a^. At this point, TC was administered as the second punch, acting as a senolytic agent to eliminate senescent tumor cells, suppress the SASP, and recover ADR-induced body weight loss. Consequently, the antitumor effect of the sequential ADR and TC treatment was stronger than that of ADR or TC alone, with lower toxicity. These results are consistent with the findings reported in a previous literature (Wang et al., 2022). Interestingly, the reduction of ADR-induced toxicity by TC treatment may also be associated with its elimination of senescent cells, which needs to be confirmed by further studies.

## 5 Conclusion

This study reveals for the first time that TC from *G. lucidum* is a novel senolytic agent, which downregulates the expression of Bcl-xl, Bcl-2, and Bcl-w proteins, reduces MMP, triggers the mitochondrial and death receptor pathways of apoptosis, and ultimately eliminates SCs. Blocking of autophagy by TC can significantly enhance the sensitivity of SCs to apoptotic signaling. TC can suppress the SASP through the inhibition of NF-κB, TFEB, P38, ERK, and mTOR signaling pathways. The sequential combination of chemotherapy with TC improves the antitumor efficacy by eliminating chemotherapy-induced senescent cells and alleviating the adverse effects of chemotherapy. Overall, we provide evidence of the use of TC to reduce ADR-associated senescence, which might help to overcome its clinical limitations in the treatment of HCC.

## Data Availability

The original contributions presented in the study are included in the article/Supplementary Material, further inquiries can be directed to the corresponding authors.
