# Supplementary figures and images for: Senolytic effect of triterpenoid complex from Ganoderma lucidum on adriamycin-induced senescent human hepatocellular carcinoma cells model in vitro and in vivo
Source: Front Pharmacol. 2024 Sep 19;15:1422363. doi: 10.3389/fphar.2024.1422363 (PMC11447279; doi:10.3389/fphar.2024.1422363)

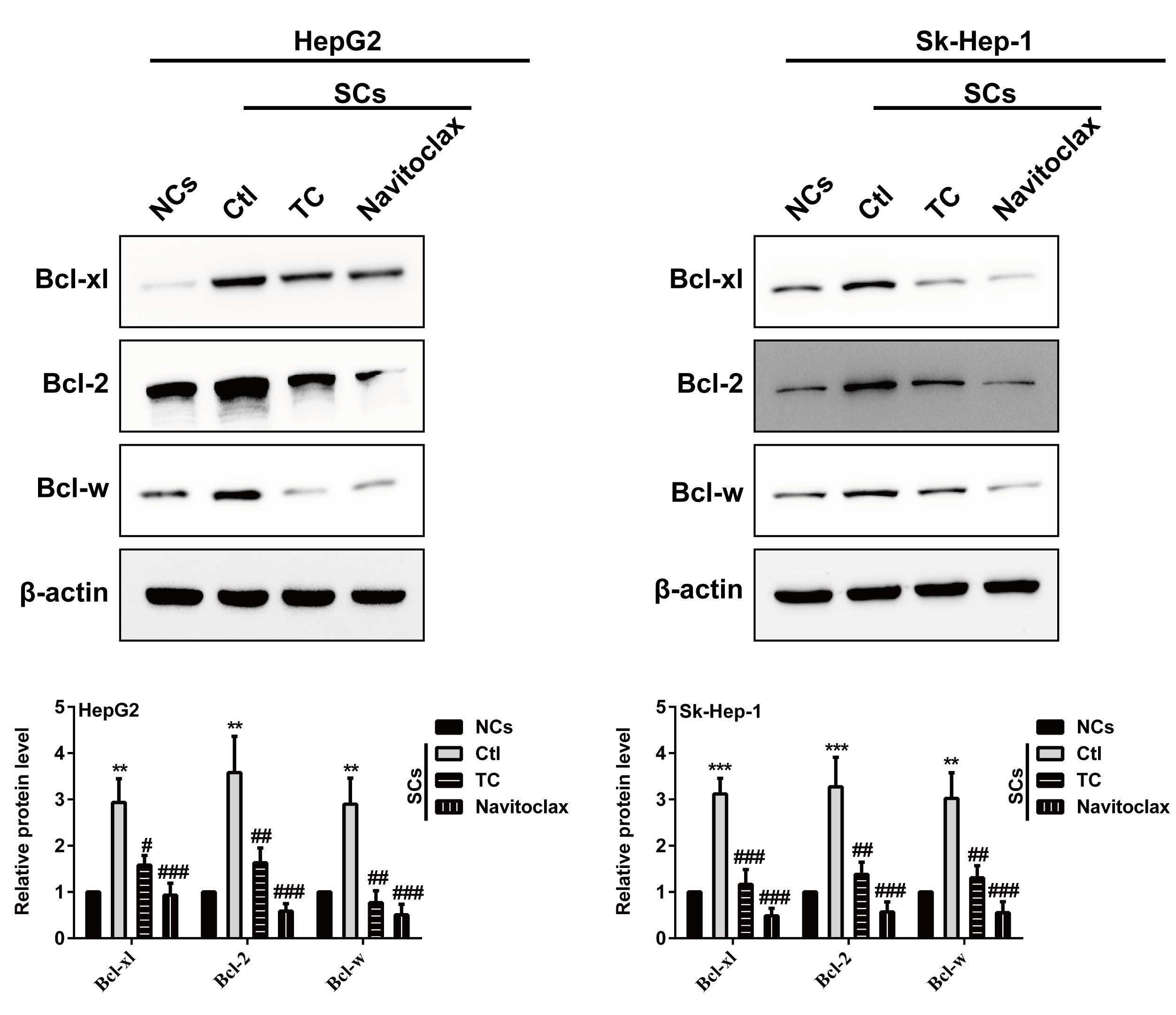

Supplement: Supplementary file 1 [file Image6.TIF]

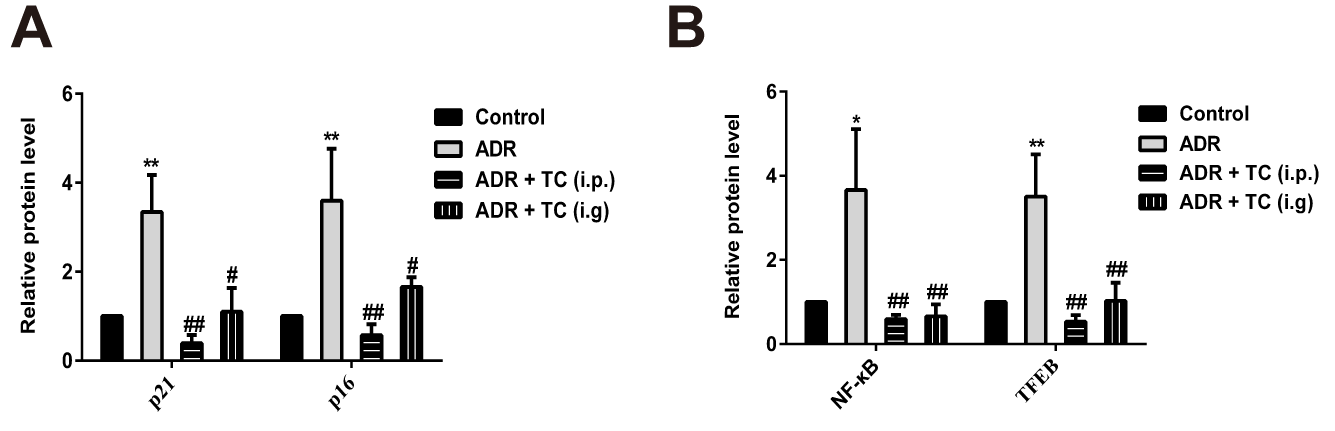

Supplement: Supplementary file 2 [file Image14.TIF]

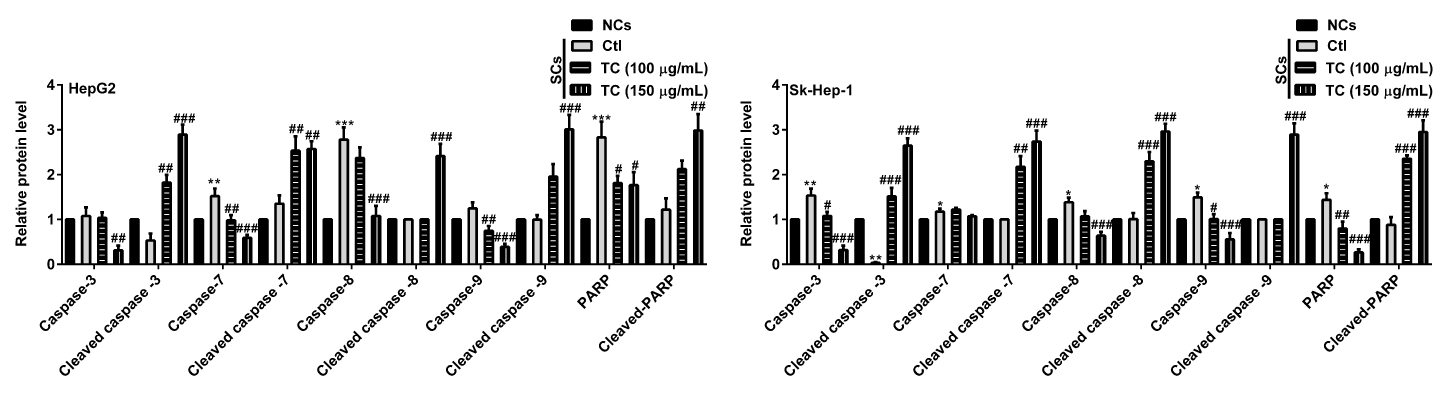

Supplement: Supplementary file 3 [file Image3.TIF]

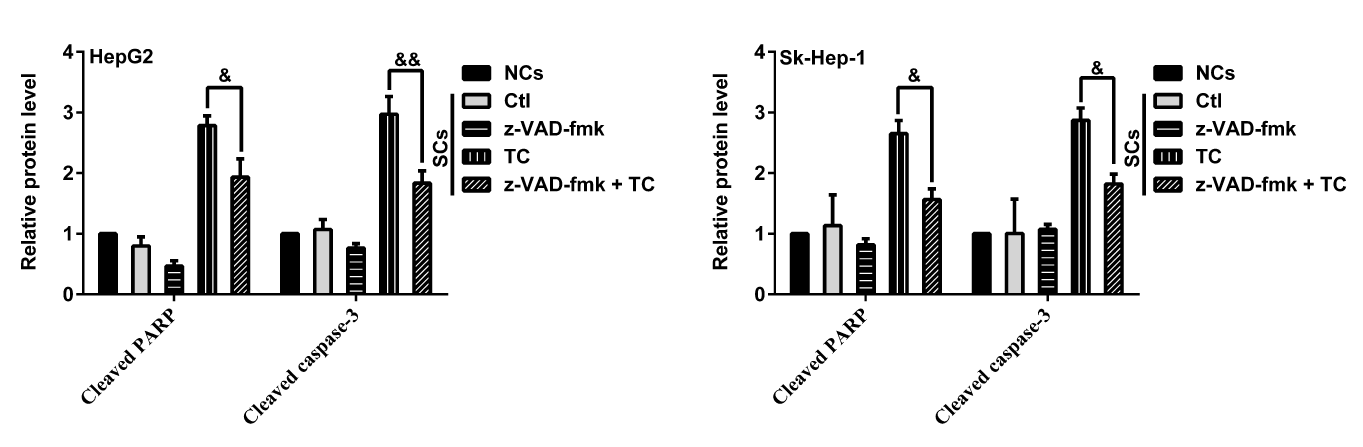

Supplement: Supplementary file 4 [file Image4.TIF]

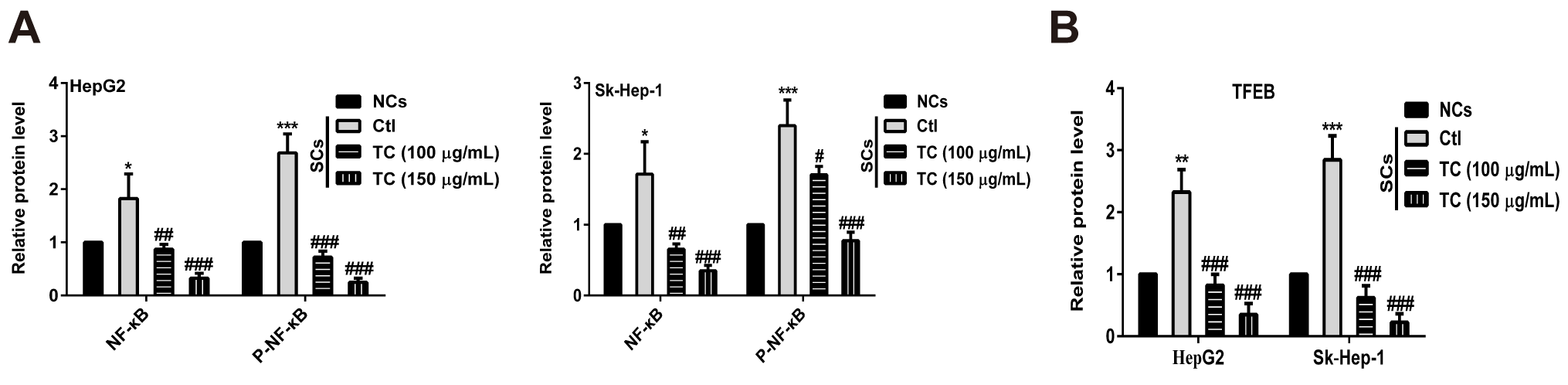

Supplement: Supplementary file 5 [file Image9.TIF]

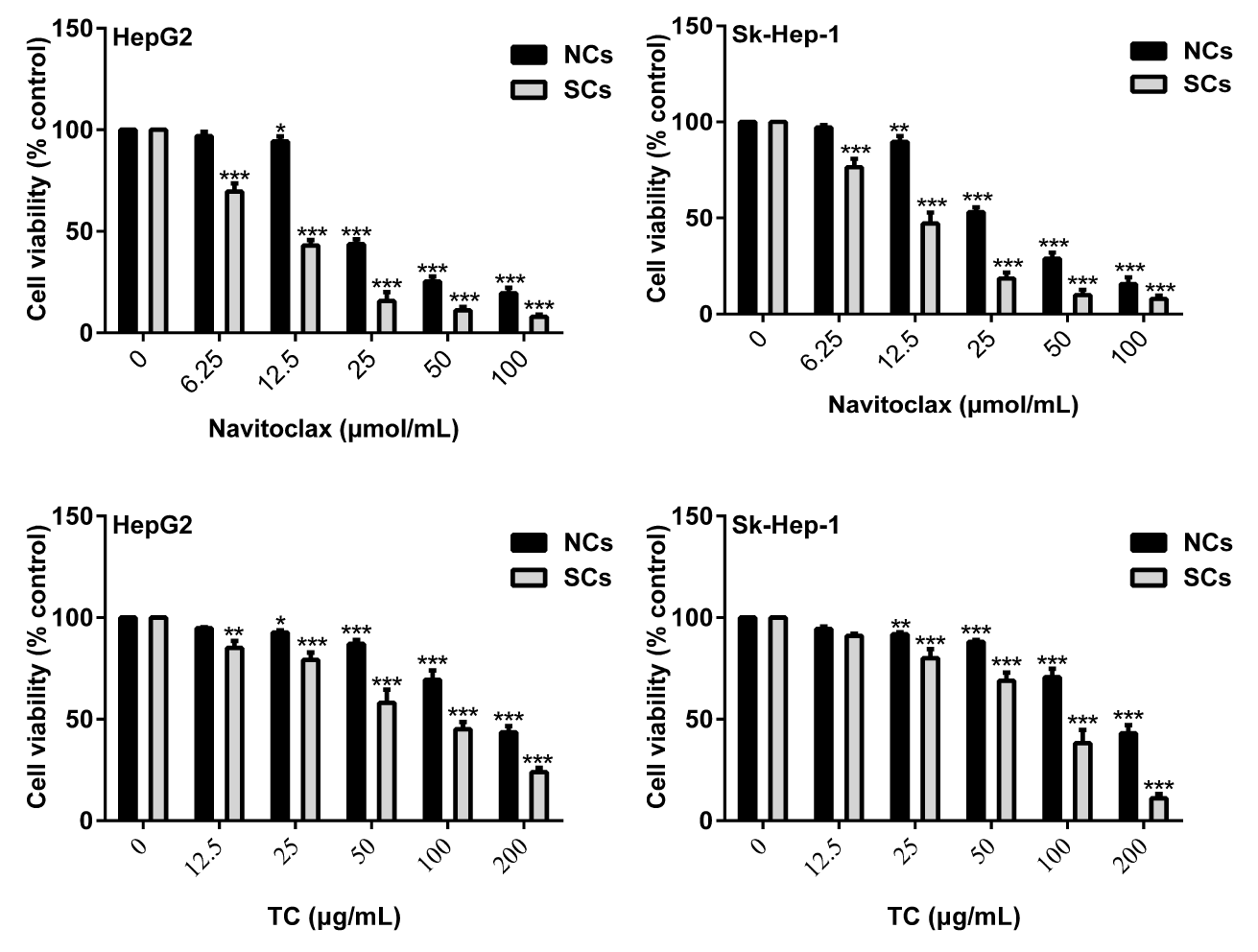

Supplement: Supplementary file 6 [file Image2.TIF]

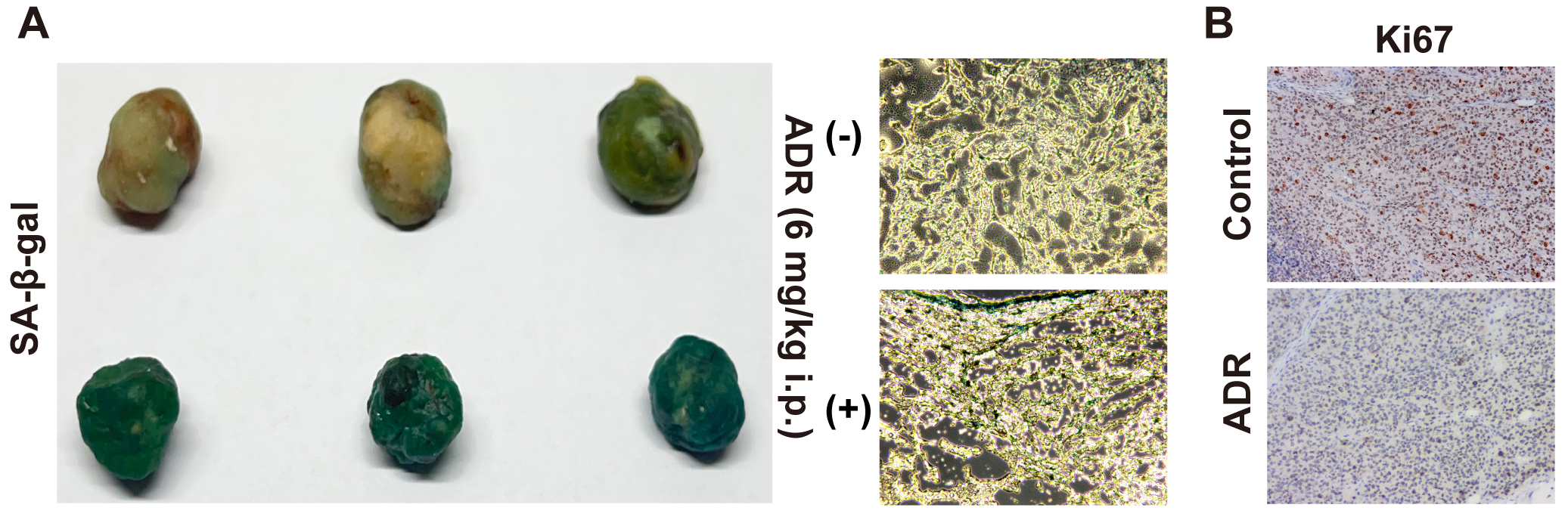

Supplement: Supplementary file 7 [file Image13.TIF]

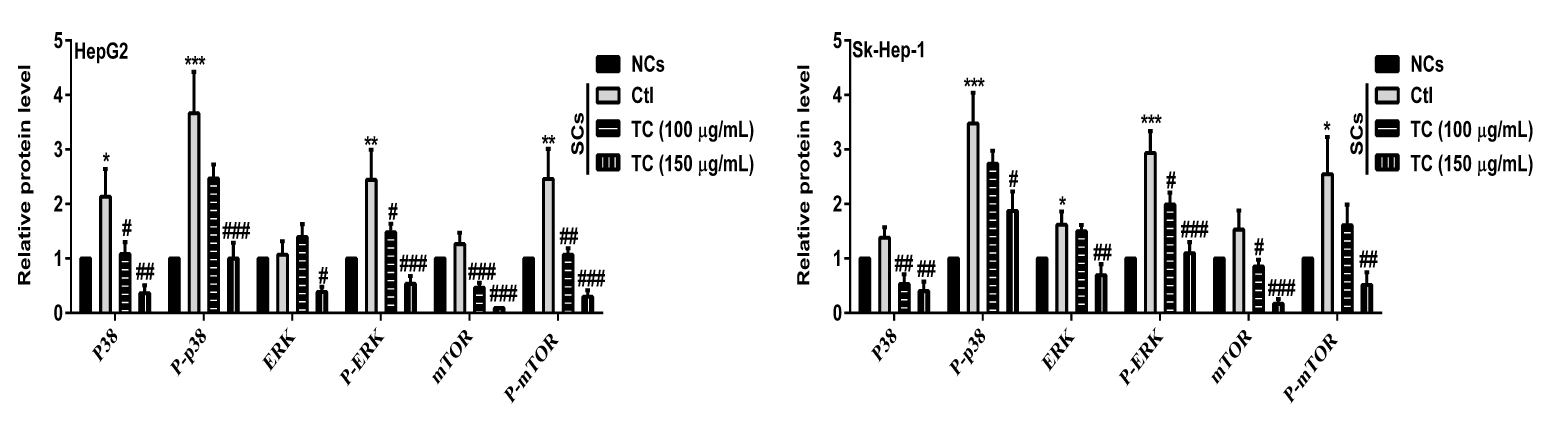

Supplement: Supplementary file 8 [file Image11.TIF]

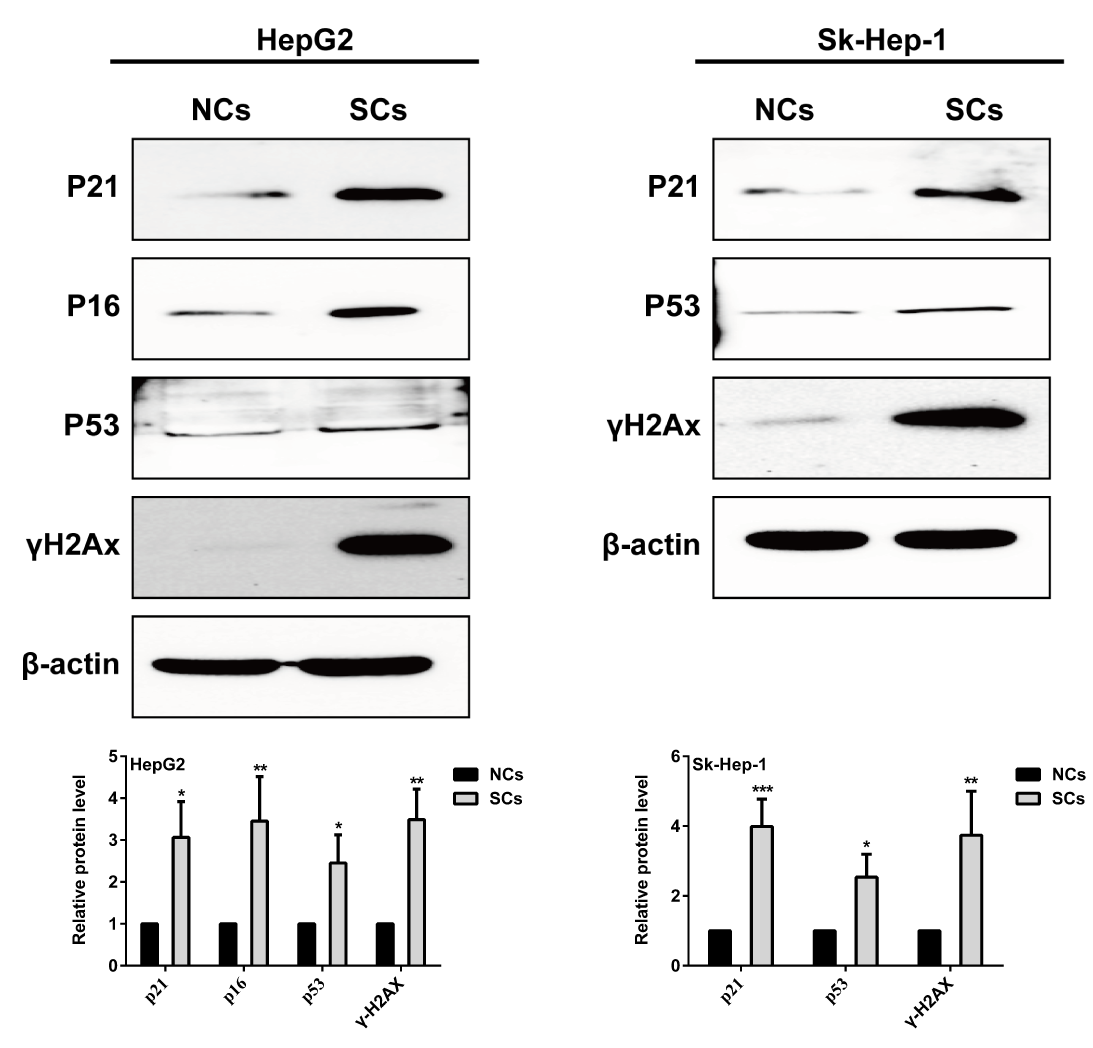

Supplement: Supplementary file 9 [file Image1.TIF]

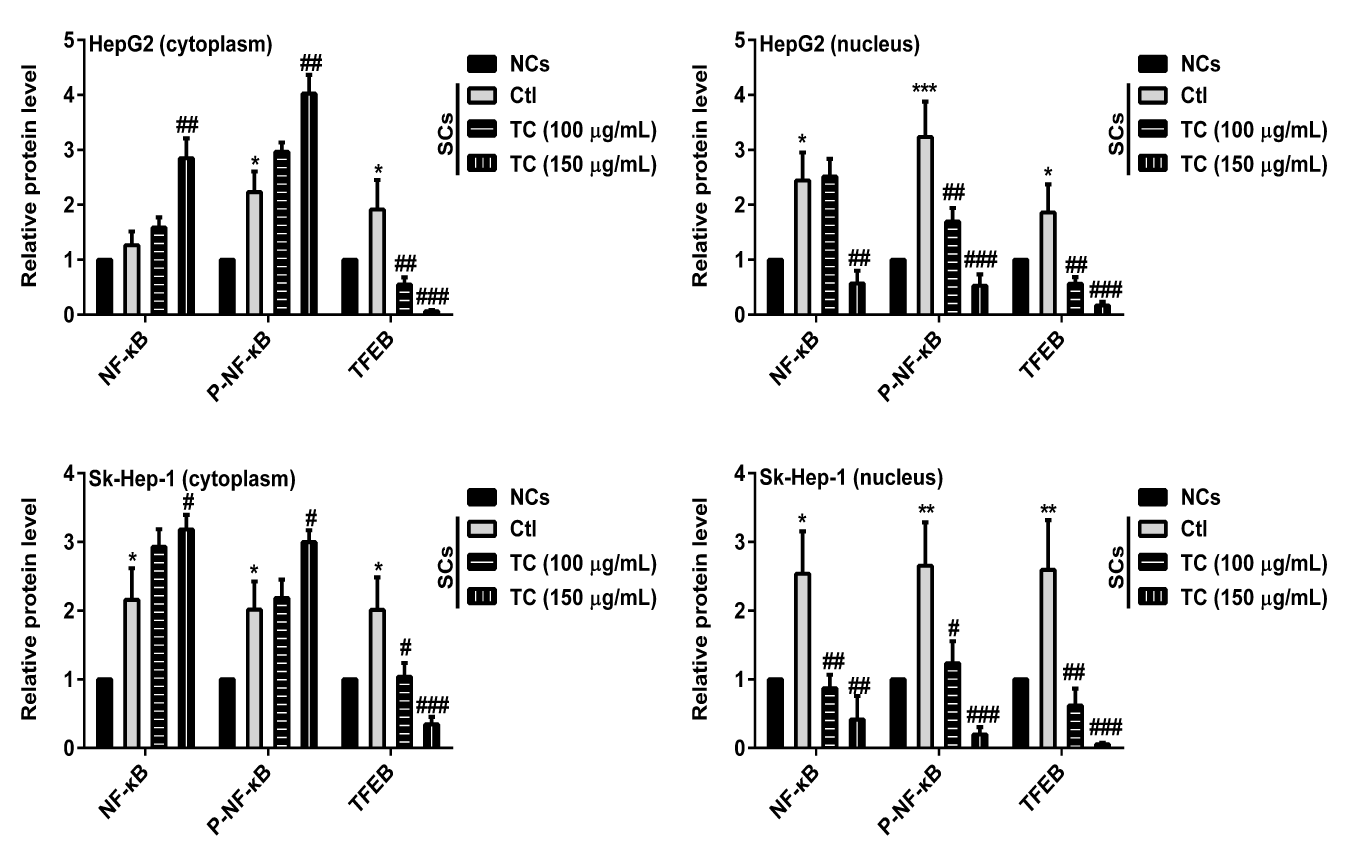

Supplement: Supplementary file 10 [file Image10.TIF]

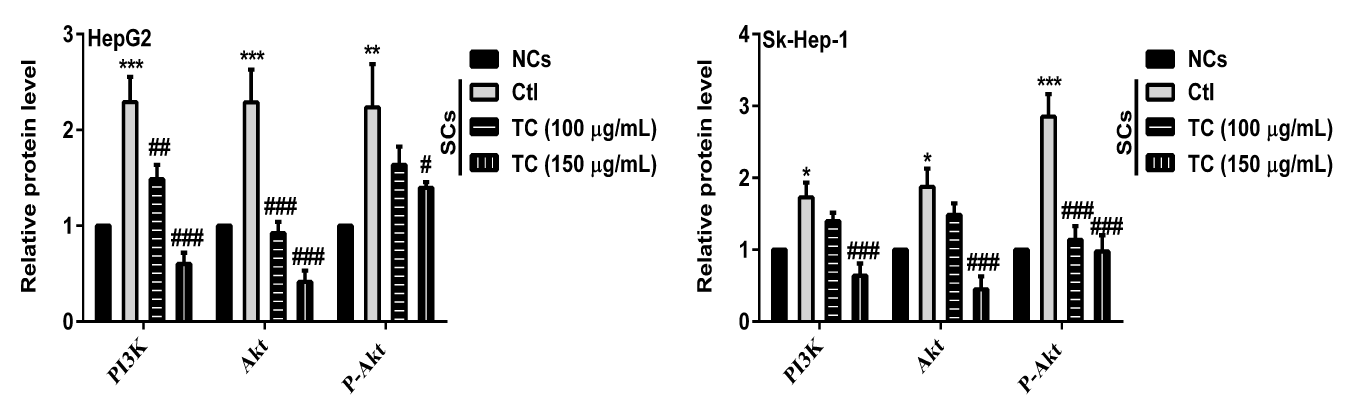

Supplement: Supplementary file 11 [file Image7.TIF]

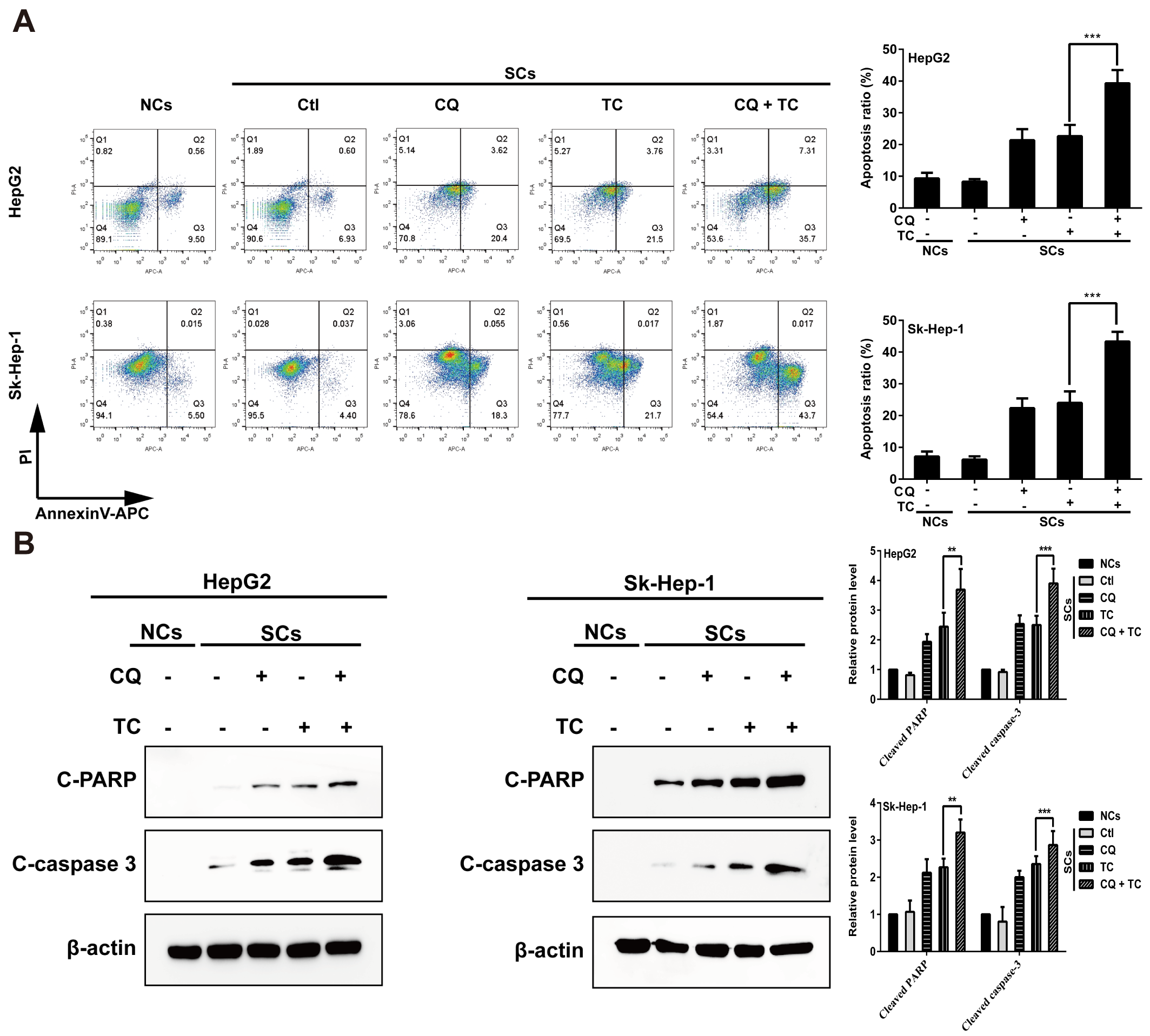

Supplement: Supplementary file 12 [file Image8.TIF]

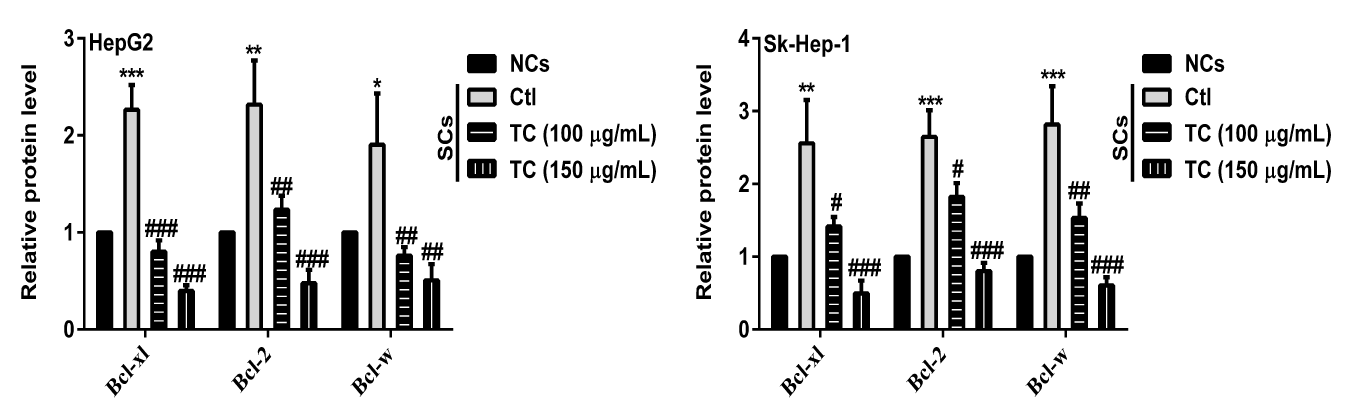

Supplement: Supplementary file 13 [file Image5.TIF]

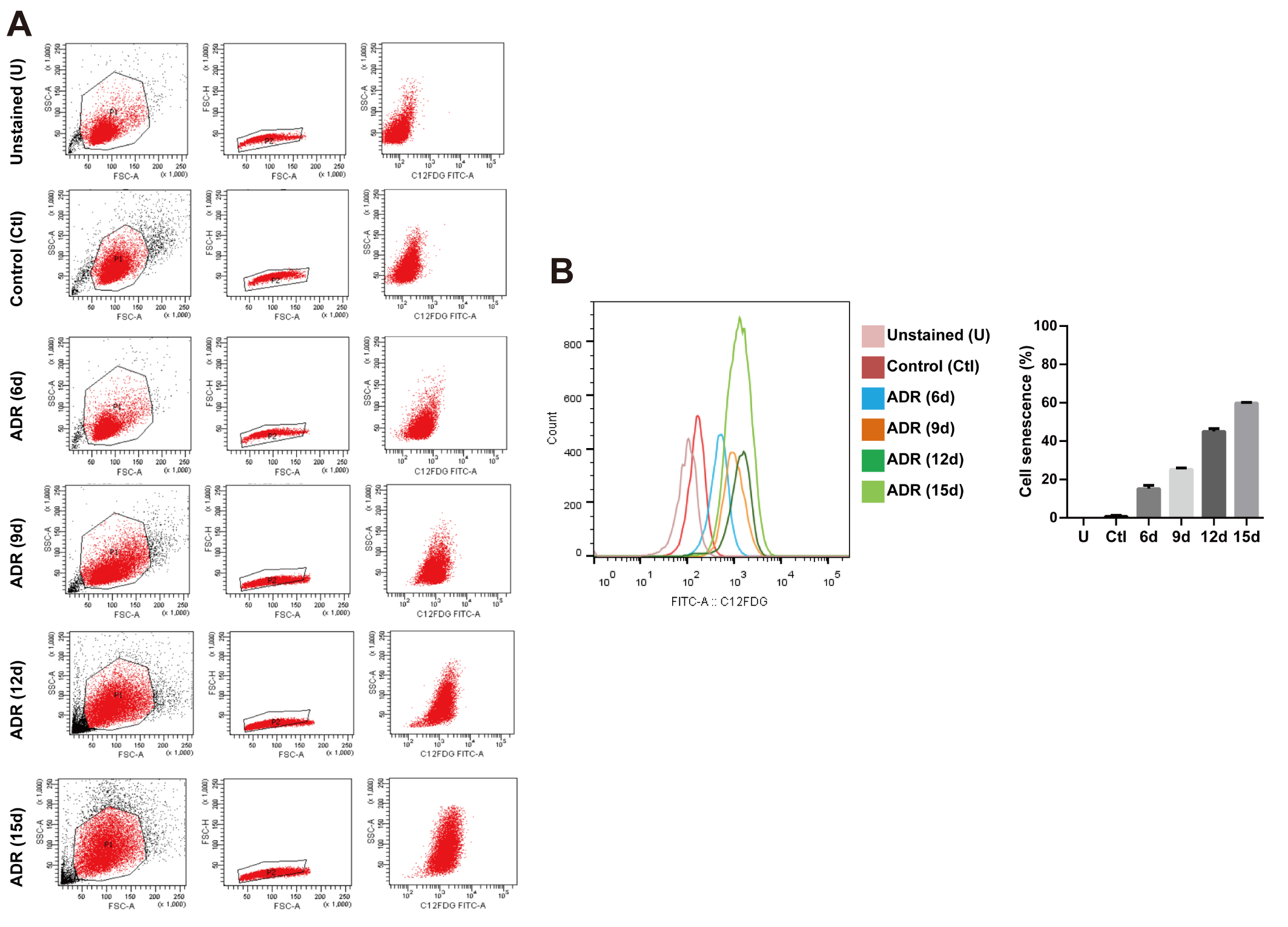

Supplement: Supplementary file 14 [file Image15.TIF]

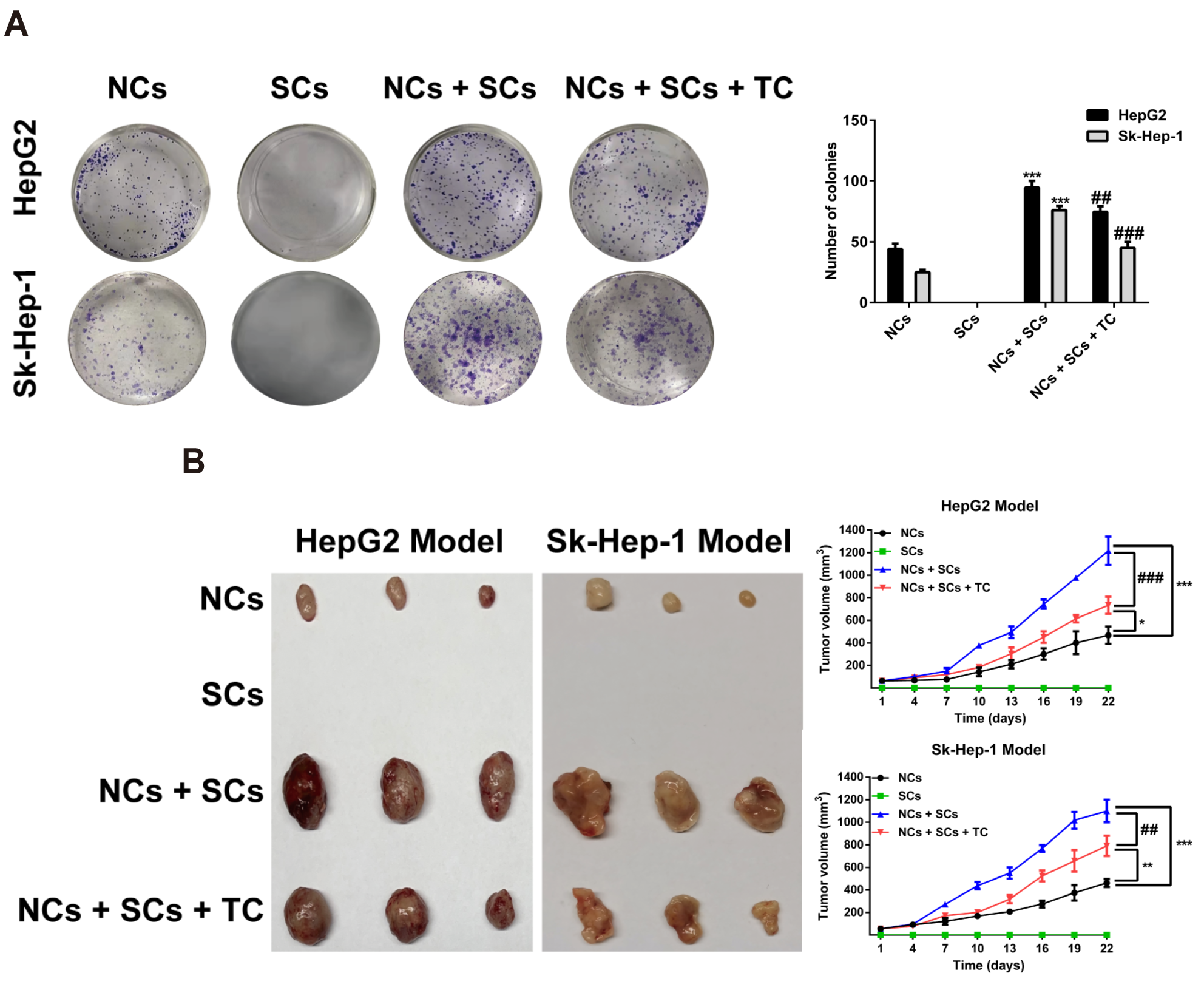

Supplement: Supplementary file 15 [file Image12.TIF]
