# Supplementary material for: Senolytic effect of triterpenoid complex from Ganoderma lucidum on adriamycin-induced senescent human hepatocellular carcinoma cells model in vitro and in vivo
Source: Front Pharmacol. 2024 Sep 19;15:1422363. doi: 10.3389/fphar.2024.1422363 (PMC11447279; doi:10.3389/fphar.2024.1422363)
Supplement: Supplementary file 16 [file DataSheet1.docx]

Supplementary Material

1. Supplementary Figures and Tables
   1. Supplementary Figures

**Supplementary Figure 1** The levels of P21^Cip1/Waf1^, p16^Ink4a^, P53, and γ-H2AX were determined by Western blotting in NCs and SCs. Data were normalized to β-actin and presented as the mean ± SD (n = 3),^*^p < 0.05, ^**^P < 0.01 and ^***^P < 0.001 versus NCs.


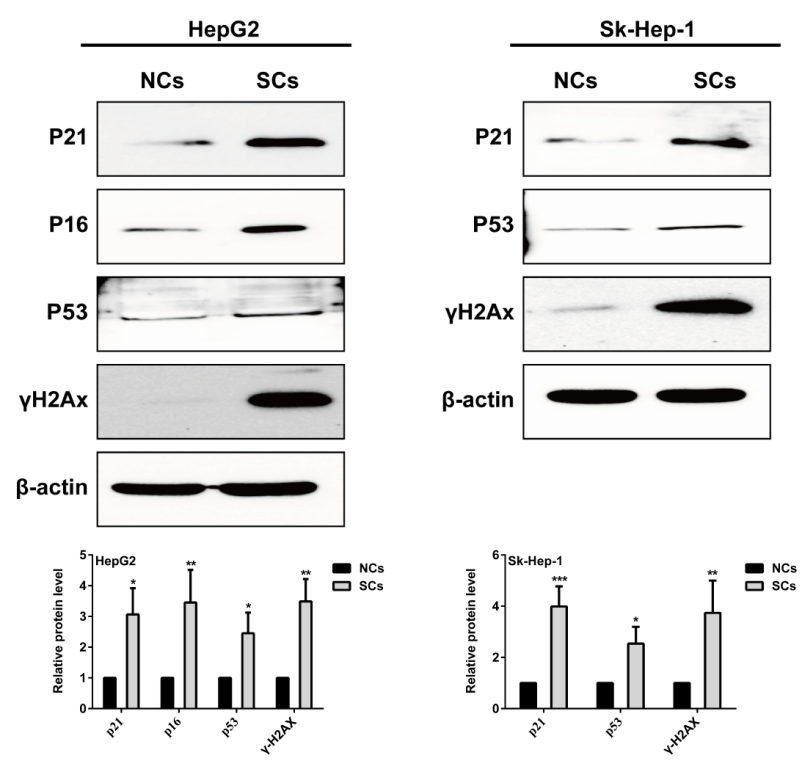


**Supplementary Figure 2** NCs and SCs were treated with various concentrations of navitoclax and TC for 48 h, and cell viability was analyzed by MTT assay. Data were presented as the mean ± SD (n = 3), ^*^p < 0.05, ^**^P < 0.01 and ^***^P < 0.001 versus the control.

**
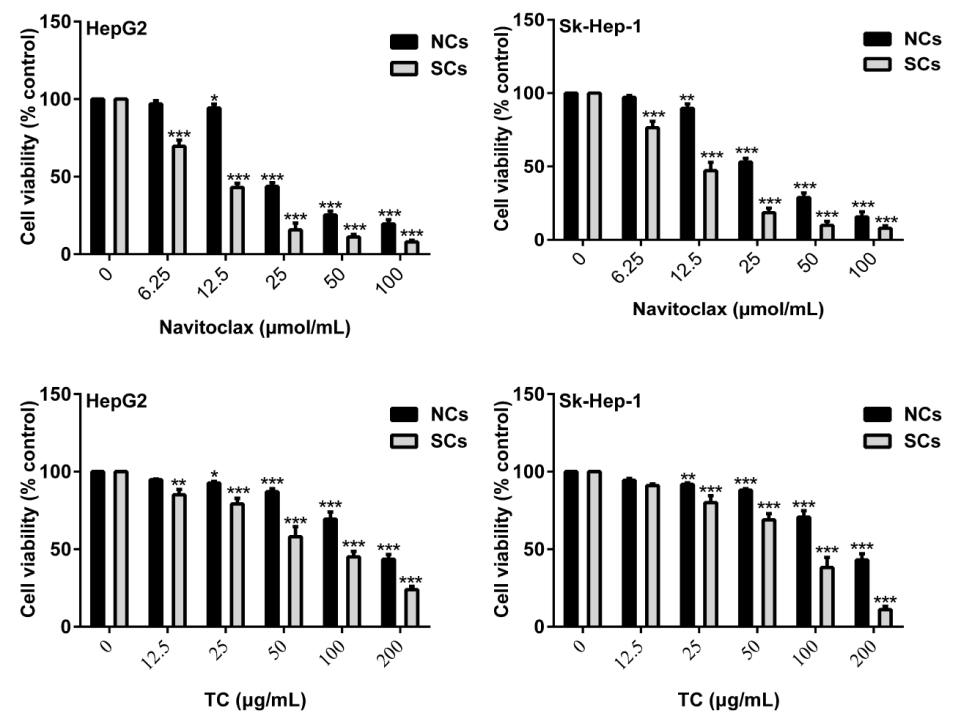
**

**Supplementary Figure 3** The quantification of western blots of Figure 3B. Data were normalized to β-actin and presented as the mean ± SD (n = 3),^*^p < 0.05, ^**^P < 0.01 and ^***^P < 0.001 versus NCs, ^#^P < 0.05, ^##^P < 0.01 and ^###^P < 0.001 versus Ctl.

**
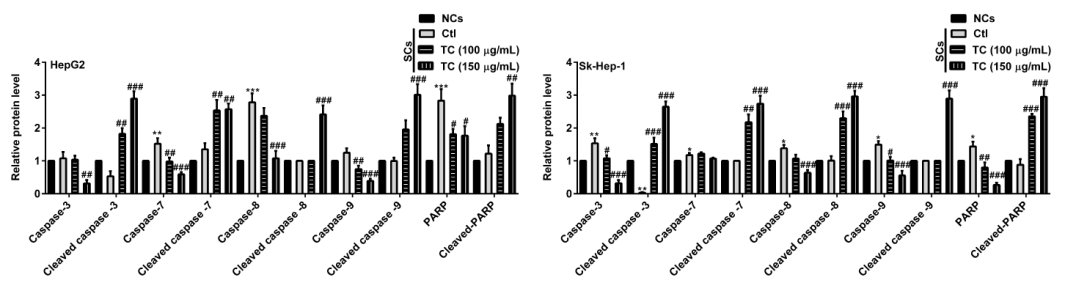
**

**Supplementary Figure 4** The quantification of western blots of Figure 3C. Data were normalized to β-actin and presented as the mean ± SD (n = 3), ^&^P < 0.05 and ^&&^P < 0.01 versus TC treatment.

**
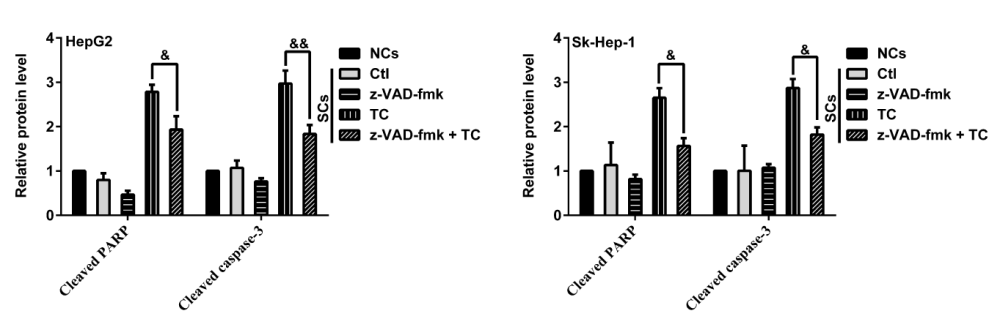
**

**Supplementary Figure 5** The quantification of western blots of Figure 3F. Data were normalized to β-actin and presented as the mean ± SD (n = 3),^*^p < 0.05, ^**^P < 0.01 and ^***^P < 0.001 versus NCs, ^#^P < 0.05, ^##^P < 0.01 and ^###^P < 0.001 versus Ctl.

**
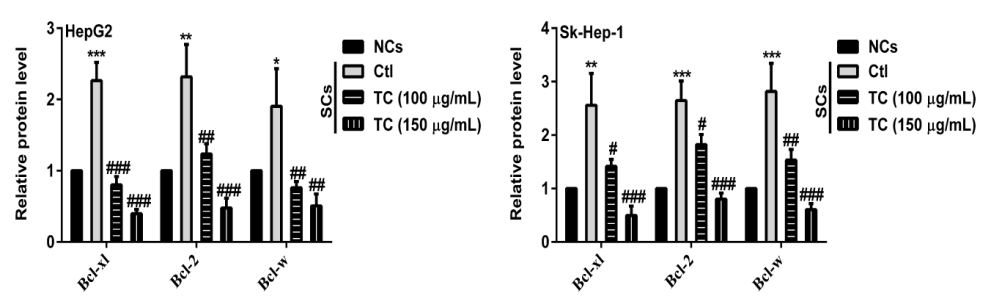
**

**Supplementary Figure 6** The expressions of Bcl-xl, Bcl-2, and Bcl-w proteins in NCs, SCs, and TC, and navitoclax-treated SCs. Data were normalized to β-actin and presented as the mean ± SD (n = 3), ^**^P < 0.01 and ^***^P < 0.001 versus NCs, ^#^P < 0.05, ^##^P < 0.01 and ^###^P < 0.001 versus Ctl.

**
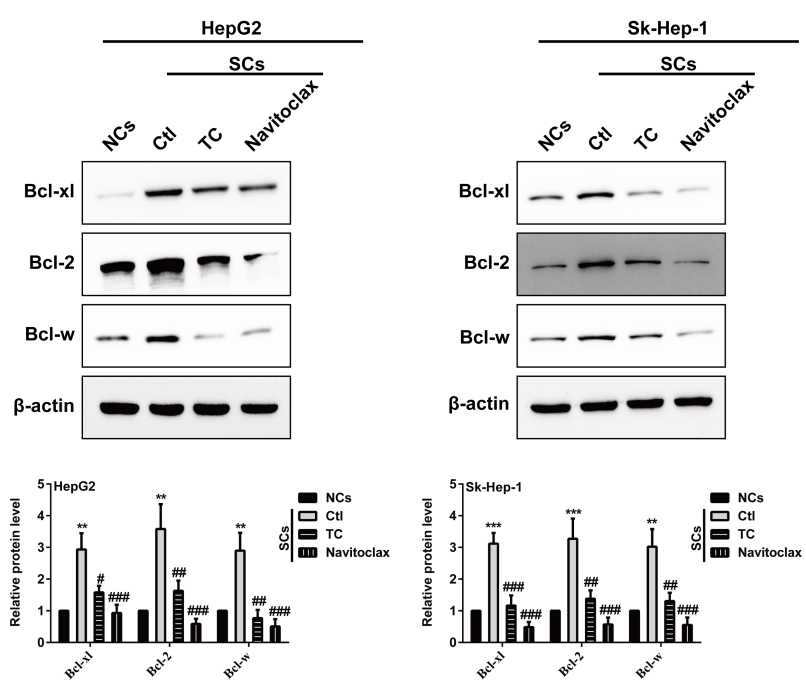
**

**Supplementary Figure 7** The quantification of western blots of Figure 3G. Data were normalized to β-actin and presented as the mean ± SD (n = 3),^*^p < 0.05, ^**^P < 0.01 and ^***^P < 0.001 versus NCs, ^#^P < 0.05, ^##^P < 0.01 and ^###^P < 0.001 versus Ctl.

**
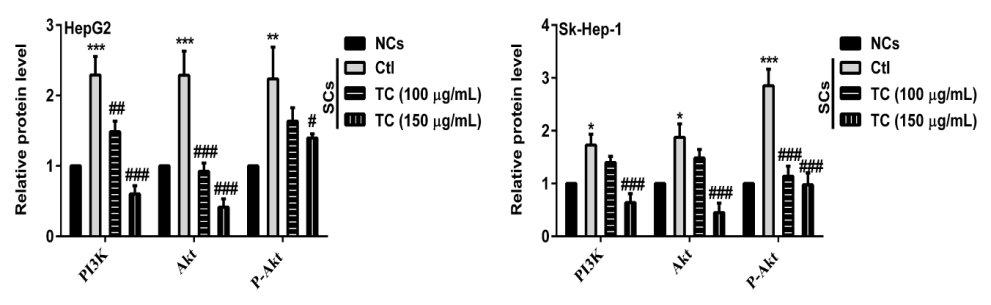
**

**Supplementary Figure 8** Autophagy blocking enhanced apoptosis induced by TC in SCs. (A) Flow cytometry analysis was used to quantify the percentage of apoptotic cells induced by CQ, TC, or a combination of them. (**B**) The expressions of cleaved PARP and cleaved caspase-3 were determined by Western blotting. Data were presented as the mean ± SD (n = 3), ^**^P < 0.01 and ^***^P < 0.001 versus TC treatment.


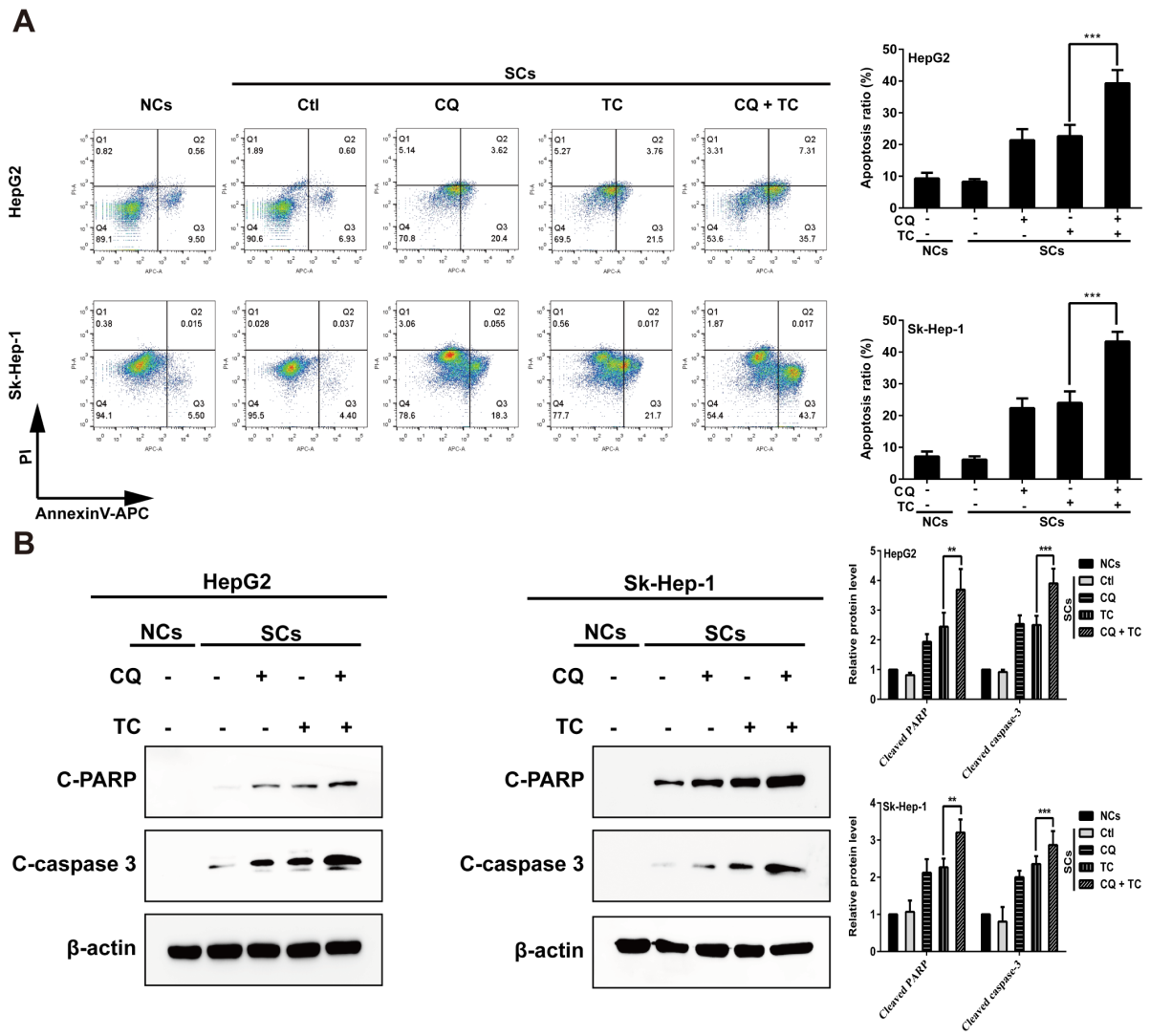


**Supplementary Figure 9** The quantification of western blots of Figures 5C and D. Data were normalized to β-actin and presented as the mean ± SD (n = 3),^*^p < 0.05,^**^P < 0.01 and ^***^P < 0.001 versus NCs, ^#^P < 0.05, ^##^P < 0.01 and ^###^P < 0.001 versus Ctl.

**
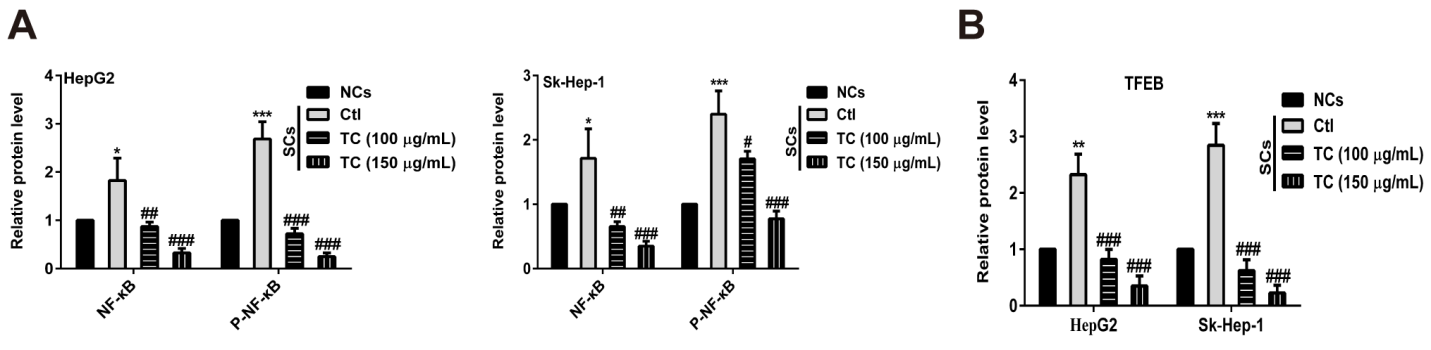
**

**Supplementary Figure 10** The quantification of western blots of Figure 5F. Data were normalized to β-actin and presented as the mean ± SD (n = 3),^*^p < 0.05, ^**^P < 0.01 and ^***^P < 0.001 versus NCs, ^#^P < 0.05, ^##^P < 0.01 and ^###^P < 0.001 versus Ctl.

**
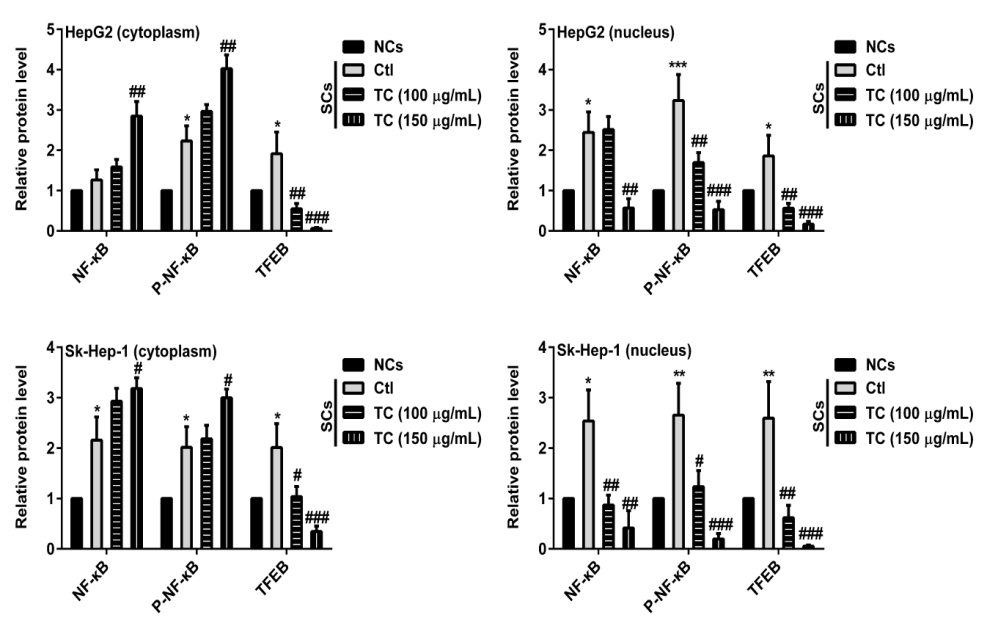
**

**Supplementary Figure 11** The quantification of western blots of Figure 5G. Data were normalized to β-actin and presented as the mean ± SD (n = 3),^*^p < 0.05, ^**^P < 0.01 and ^***^P < 0.001 versus NCs, ^#^P < 0.05, ^##^P < 0.01 and ^###^P < 0.001 versus Ctl.

**
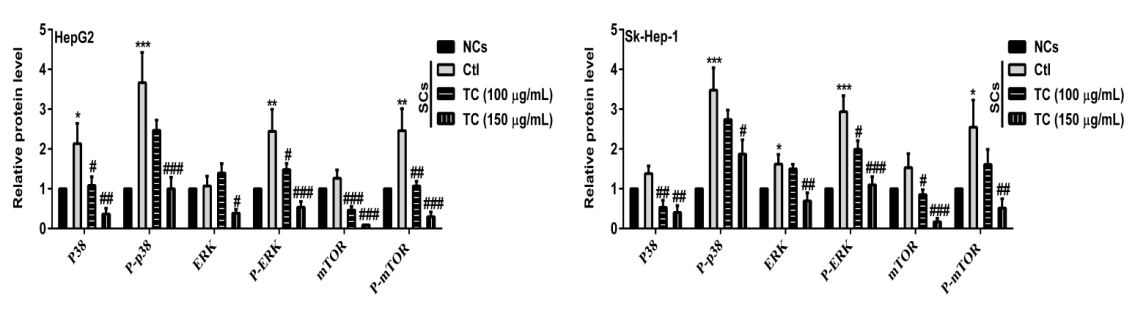
**

**Supplementary Figure 12** TC attenuated the growth stimulation induced by SCs *in vitro* and *in vivo* (**A**) The colony formation assay was used to detect the growth stimulation effect of SCs on NCs in the absence and presence of TC-treated SCs. (**B**) Stimulation of tumor growth was measured in nude mice injected with NCs and SCs alone or in combination in the absence and presence of TC-treated SCs. Data were presented as the mean ± SD, ^*^P < 0.05, ^**^P < 0.01 and ^***^P < 0.001 versus NCs, ^##^P< 0.01 and ^###^P< 0.001 versus NCs + SCs group.


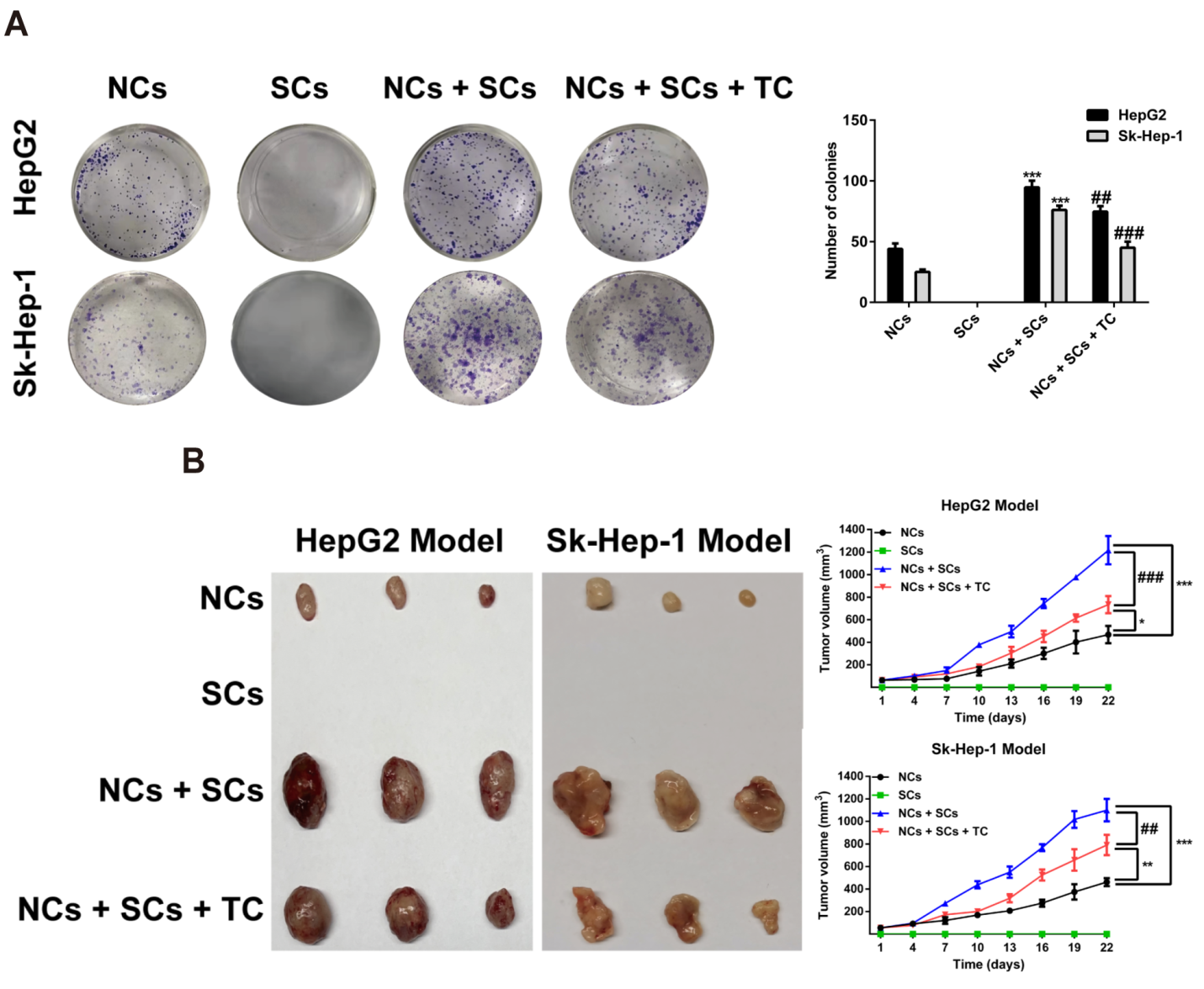


**Supplementary Figure 13** ADR induced *in vivo* senescence in HepG2 xenograft model. (A) SA-β-Gal staining of control and ADR-induced senescence in tumors and frozen sections from HepG2 xenograft. (**B**) Ki67 staining in frozen sections from HepG2 xenograft treated with vehicle or ADR.


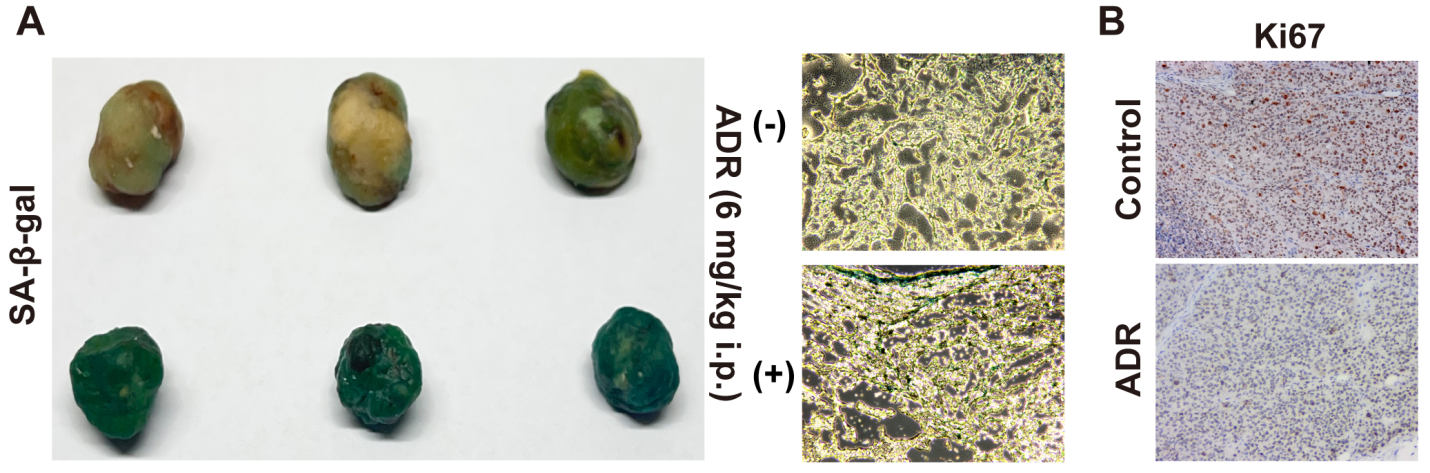


**Supplementary Figure 14** The quantification of western blots of Figures 6G and K. Data were normalized to β-actin and presented as the mean ± SD (n = 3), ^*^P < 0.05 and ^**^P < 0.01 versus the control，^#^P < 0.05 and ^##^P < 0.01 versus ADR treatment.

**
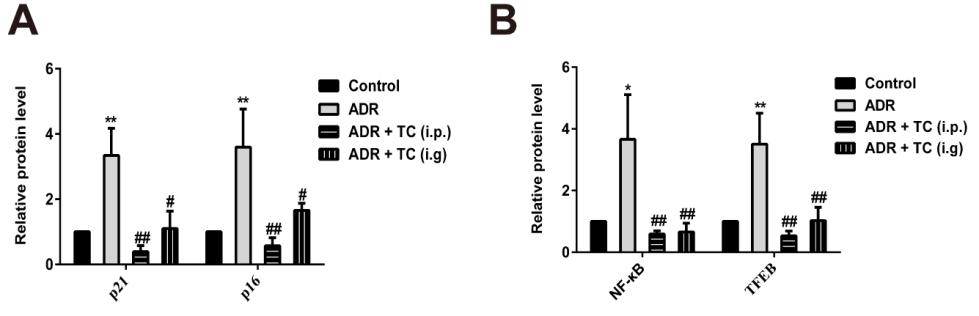
**

**Supplementary Figure 15** The quantification of SCs in PBMCs following ADR treatment at different time points by flow cytometry. (A) Gating data plots for PBMCs to identify SCs. (B) Histogram and analysis of senescent subsets of PBMCs from control and ADR-treated animals.


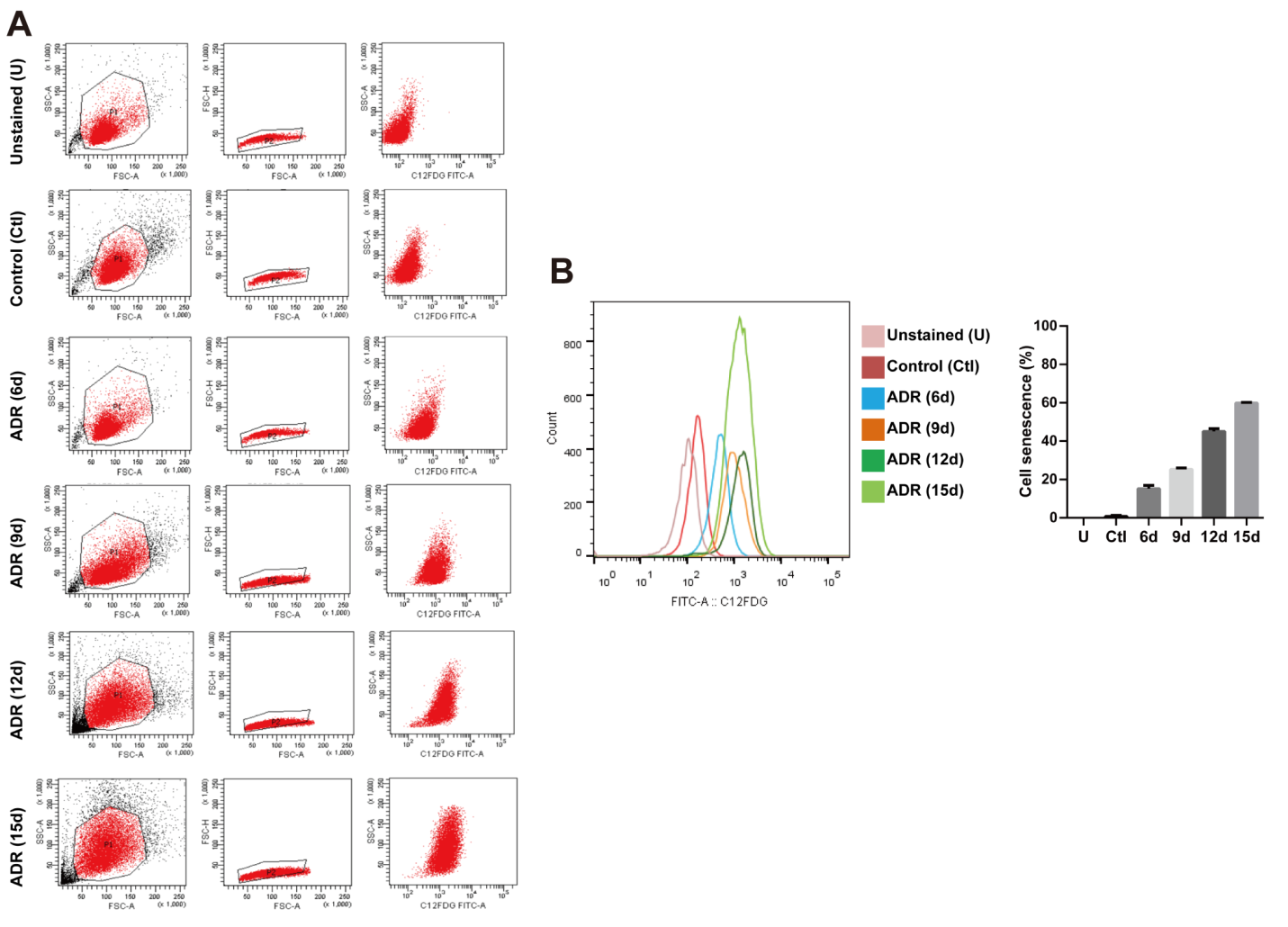


- 1. Supplementary Tables

**Supplementary Table 1** One-two punch scheme of ADR and TC for HCC xenograft model

| Groups | Treatment | Tumor growth | First punch | Second punch |
| --- | --- | --- | --- | --- |
| a | saline | 100 mm^3^ | 2 weeks | 3 weeks |
| b | TC 100 mg/kg/day, i.p. | 100 mm^3^ | － | 3 weeks |
| c | TC 250 mg/kg/day, i.g | 100 mm^3^ | － | 3 weeks |
| d | ADR 6 mg/kg/week, i.p. | 100 mm^3^ | 2 weeks | － |
| e | ADR 6 mg/kg/week, i.p. | 100 mm^3^ | 2 weeks | － |
|  | TC 100 mg/kg/day, i.p. | 100 mm^3^ | － | 3 weeks |
| f | ADR 6 mg/kg/week, i.p. | 100 mm^3^ | 2 weeks |  |
|  | TC 250 mg/kg/day, i.g | 100 mm^3^ | － | 3 weeks |
